# Keys to the blow flies of Taiwan, with a checklist of recorded species and the description of a new species of *Paradichosia* Senior-White (Diptera, Calliphoridae)

**DOI:** 10.3897/zookeys.434.7540

**Published:** 2014-08-14

**Authors:** Shih-Tsai Yang, Hiromu Kurahashi, Shiuh-Feng Shiao

**Affiliations:** 1 Department of Entomology, National Taiwan University Taipei, Taiwan; 2 Department of Medical Entomology, National Institute of Infectious Diseases, Tokyo, Japan

**Keywords:** Calliphoridae, diagnostic key, new species, taxonomy

## Abstract

Blow flies (Diptera: Calliphoridae) show a great diversity in behavior and ecology, play important roles in ecosystems, and have medical and forensic importance to humans. Despite this, the taxonomy and classification of Taiwan's Calliphoridae have rarely been studied. In this study, specimens of Taiwanese calliphorids were collected and carefully studied, and all 76 species recorded in Taiwan are listed following the identification keys. Dichotomous keys to all subfamilies, tribes, genera, and species of blow flies recorded in Taiwan are provided, including 16 species that are newly recorded from Taiwan. In addition, one new species of the genus *Paradichosia* Senior-White is described and illustrated. We also discuss the morphological differences between the specimens of *Silbomyia hoeneana* Enderlein collected from China and Taiwan, a species that has only been found previously in Southern China.

## Introduction

Calliphorids show great diversity in behavior and ecology. Some species parasitize terrestrial vertebrates and invertebrates such as insects, snails, amphibians, mammals, and others rely on animal carcasses or excrement for survival (Kano and Shinonaga 1968). Some calliphorid species are regarded as serious pests because they carry pathogens or parasitize livestock or humans, causing myiasis (Tumrasvin et al. 1979; Sawabe et al. 2006). Other species called carrion flies are considered important scavengers due to their necrophagous feeding behaviors (Singh et al. 1979). Some species also provide an alternative way to estimate the minimum post-mortem interval of a victim or an animal in forensic investigations (Catts and Goff 1992). Adult blow flies often visit flowers, and some species like *Chrysomya megacephala* (Fabricius) are pollinators of fruits (Dag and Gazit 2001). The Calliphoridae are thus important in many different fields. In this article, keys to the subfamilies, tribes, genera, and species recorded from Taiwan are provided, hopefully to help in the understanding and identification of these useful flies in studies of medical entomology, forensic entomology, agricultural pollination, and other related fields.

Only fragmentary studies have been done on the taxonomy of Taiwanese Calliphoridae. Hennig (1941) listed 46 species of Calliphoridae from Taiwan. James (1977) mentioned 34 Taiwanese species in “A Catalog of Diptera of the Oriental Region.” Kurahashi (1987a) published a key to the species of Taiwan’s Calliphorini and Luciliini tribes. Fan (1992) published a key to the common calyptrate flies of China, including 40 Taiwanese species. Recently, a new *Lucilia* species, *Lucilia taiwanensis*, was described by Kurahashi and Kano (1995).

## Materials and methods

Adult specimens of Taiwanese calliphorids examined in this study were collected and pinned by the authors and colleagues, and additional specimens were collected by K. Kanmiya, R. Kano, K. Matsuki, and S. Ueno. Systematics and nomenclature adopted in this paper mainly follows Rognes (1991, 1997, 2002, 2009a, 2009b, 2011a), Fan (1992), and Kurahashi and Bunchu (2011). Specimens collected by the first author were mostly deposited in the Insect Museum of National Taiwan University (NTU). Other specimens were deposited in the National Museum of Nature and Science, Tsukuba (NSMT), Bishop Museum, Honolulu (BPBM), Carnegie Museum of Natural History, Pittsburgh (CMNH), and International Department of Dipterology, Tokyo (IDD). Some specimens are personal collection of Mr. K. Harusawa (PCKHa) as noted in the text. The terminology used in this study mainly follows that of Senior-White et al. (1940), Fan (1992), Triplehorn and Johnson (2005). Frons measurement and the calculation of the frons index follows Fan (1992).

## Results and discussion

Examined specimens are classified into nine subfamilies: Ameniinae, Bengaliinae, Calliphorinae, Chrysomyinae, Luciliinae, Melanomyinae, Rhiniinae, Phumosiinae, and Polleniinae. In total, 76 calliphorid species are listed herein based on our direct examinations and the literature. Among them, 16 species are newly recorded from Taiwan, with an asterisk (*) preceding each of them in the checklist. The names in this checklist include mainly the species examined in this research and partially also the species recorded in the literature. Some species that have not yet been recorded in Taiwan are also included in this key with a footnote [e.g., ‘not recorded from Taiwan’]. We believe these latter species have the potential to be found in future Taiwan surveys because they occur in nearby areas of the Orient.

Widespread Oriental elements share the highest proportion (53.9%, 41/76) of Taiwan’s calliphorid fauna, while endemic species have the second-highest (15.8%, 12/76). Australasian and Oriental-Australasian elements share 14.5% (11/76), cosmopolitan elements 6.6% (5/76), pantropical elements 2.6% (2/76), Holarctic species 1.3% (1/76), and Palaearctic species 5.3% (4/76).

## Practical keys

### Key to the subfamilies of Taiwanese Calliphoridae

**Table:** 

1	Posterodorsal side of R stem vein of wing setose	2
–	Posterodorsal side of R stem vein of wing not setose	3
2	Upper occiput with setulae and pollinose, without glossy submarginal band; lower facial margin not protruding much; prealar knob with erect hairs (except for tribe Phormiini); thoracic squama hairy on dorsal surface, largely lobulate, subtruncate at apex, concave on outer margin	Subfamily CHRYSOMYINAE
–	Upper occiput often without setulae, with bare glossy submarginal band; lower facial margin often protruding in front of vibrissal corners obviously; prealar knob bare or pubescent, without distinct hairs; thoracic squama (lower calypter) bare or pubescent on dorsal surface, usually tongue-like, narrowly rounded at apex, straight on outer margin, rarely lobulate in some species of *Isomyia*	Subfamily RHINIINAE
3	Anterior lappet of metathoracic spiracle with conspicuous backward-pointing tuft of long hairs; postscutellum forming definite convex swelling which is micro-rugose and sometimes shows a slight trace of a shallow median incision; female postabdomen non-telescopic, modified for deposition of grown larvae; large tachinid-like flies	Subfamily AMENIINAE
–	Anterior lappet of metathoracic spiracle bare or at most with very few inconspicuous hairs; postscutellum not at all convex or at most with a rudimentary trace of swelling, not as above; female postabdomen forming telescopic ovipositor, but sometimes short in larviparous species for deposition of first instar larvae	4
4	Propleuron hairy	5
–	Propleuron bare	8
5	Posterior part of suprasquamal ridge with tuft of setulose erect hairs (posterior parasquamal tuft) on small well-defined black sclerite	Subfamily LUCILIINAE
–	Posterior parasquamal tuft absent	6
6	Supraspiracular convexity clothed with long, upstanding fine hairs; anterior part of suprasquamal ridge bare; distance between right and left presutural acrostichal bristles (*ac*) rather large, almost equal to distance between presutural *ac* and dorsocentral bristles (*dc*); mesothoracic spiracle rather large, sometimes swollen; thoracic squama bare	Subfamily PHUMOSIINAE, *Caiusa* Surcouf
–	Supraspiracular convexity bare or pubescent	7
7	Hairs on thoracic squama widely distributed	Subfamily CALLIPHORINAE
–	Thoracic squama bare, or only few hairs fragmentarily distributed	Subfamily MELANOMYINAE
8	Prosternum usually hairy; thorax not clothed in golden curly hairs; eyes dichoptic, eyes widely separated in both male and female; proboscis short and stout, boat-shaped; body at least partly yellowish	Subfamily BENGALIINAE, *Bengalia* Robineau-Desvoidy
–	Prosternum bare; thorax usually with golden curly hairs, sometimes lacking; head usually holoptic or subholoptic in males, sometimes dichoptic	Subfamily POLLENIINAE

### Subfamily AMENIINAE

#### Key to the tribes and genera of AMENIINAE

**Table:** 

1	Head almost always with a very large facial carina; propleuron and prosternum almost always hairy; hind tibia with apical posteroventral bristle (*pv*); outer posthumeral bristle (*ph*) situated mesad of presutural bristle (*prs*); ventral surface of costa setulose between apices of subcostal (Sc) and first longitudinal (R_1_) veins	Tribe Ameniini, *Silbomyia* Macquart
–	Head without facial carina; propleuron bare; prosternum bare, sometimes hairy; hind tibia without apical *pv*; outer *ph* situated laterad of *prs*; ventral surface of costa bare between apices of veins Sc and R_1_	Tribe Catapicephalini, *Catapicephala* Macquart

#### Tribe Ameniini

##### Key to the species of *Silbomyia*

**Table:** 

1	Facial carina longer and distinctly fusiform, slightly longer than distance from lunule to anterior ocellus; male antennae longer than in female, third segment of antenna (AS_3_; first flagellomere) in male at least 4 times as long as AS_2_ (pedicel); postorbits yellow or silvery yellow	*Silbomyia cyanea* (Matsumura)
–	Facial carina short, and roof-like, shorter than distance from lunule to anterior ocellus; antennae about equal in length in both sexes, AS_3_ not more than 3.9 times as long as AS_2_; postorbits silvery white	2
2	One or both of third tergite (T_3_) and T_4_ almost always with one pair or more of strong median discal setae; color usually dark blue to violet, sometimes green; male frontal stripe (interfrontal area) no more than 3 times as wide as one parafrontal; distance between eyes at lower margin subequal or slightly broader than that at vertex in frontal view in male; lobe of fifth sternite (ST_5_) shorter than basal part	*Silbomyia sauteri* Enderlein
–	Third tergite and T_4_ without or only T_3_ with one pair of very weak discal setae; color green to blue; male interfrontal area about 3.5 times as wide as one parafrontal; distance between eyes at lower margin slightly narrower than that at vertex in frontal view in male; lobe of ST_5_ longer than basal part	*Silbomyia hoeneana* Enderlein

#### Tribe Catapicephalini

##### Key to the species of *Catapicephala*

**Table:** 

1	Eyes hairy	*Catapicephala dasyophthalma* Villeneuve
–	Eyes bare	2
2	Antenna orange; tomentum on face golden	*Catapicephala ruficornis* Villeneuve
–	Antenna entirely fuscous; tomentum on face grey; T_4_, T_5_ brilliant metallic blue; silver white pollinosity on lateral and ventral sides of T_5_ extending to dorsal side, visible in dorsal view; fronto-orbital bristles (*ors*) 0+1 in male, 2+1 in female	*Catapicephala splendens* Macquart

### Subfamily CALLIPHORINAE

#### Key to the genera of CALLIPHORINAE

**Table:** 

1	Presutural *ac* usually 1, rarely absent; facial carina more or less developed	*Polleniopsis* Townsend
–	Presutural *ac* 2; facial carina absent	2
2	Head dichoptic in both male and female; AS_3_ elongate, more than 4 times as long as AS_2_; presutural intra-alar bristles (*ia*) absent	*Tainanina* Villeneuve
–	Head holoptic or subholoptic in male, dichoptic in female; AS_3_ variable in length; presutural *ia* present or absent	3
3	Presutural *ia* absent; eyes in male subholoptic; male hypopygium prominently swollen, metallic	*Aldrichina* Townsend, *Aldrichina grahami* (Aldrich)
–	Presutural *ia* present; eyes in male holoptic; male hypopygium not remarkably developed	4
4	Body small- to medium-sized; distance between rows of right and left presutural *ac* rather small (*fig. 1b* in Kurahashi 1970); male paraphallus with wide, sickle-like tip	*Bellardia* Robineau-Desvoidy (Kurahashi (1987b) treat it in genus *Onesia*)
–	Body large-sized; distance between rows of right and left presutural *ac* rather large (*fig. 1a* in Kurahashi 1970); male paraphallus with slender tip	*Calliphora* Robineau-Desvoidy

#### Key to the species of *Calliphora*

**Table:** 

1	Postgena clothed with orange or pale yellow hairs, or intermixed with black ones; mesothoracic spiracle fuscous black	*Calliphora vomitoria* (Linnaeus)
–	Postgena clothed wholly with black hairs	2
2	Mesothoracic spiracle yellowish brown to orange	*Calliphora nigribarbis* Vollenhoven
–	Mesothoracic spiracle black or blackish brown	*Calliphora pattoni* Aubertin

#### Key to the species of *Bellardia*

**Table:** 

1	Body length less than 6 mm; R_5_ cell closed at wing margin; vein M_1+2_ gently curved, with dull angle	*Bellardia menechma* (Séguy)
–	Body length longer than 8 mm; R_5_ cell open at wing margin; vein M_1+2_ bent with a right angle	*Bellardia pubescens* (Macquart)

#### Key to the species of *Tainanina*

**Table:** 

1	Fronto-orbital bristles 0 in male, 2+1 in female	*Tainanina sarcophagoides* (Malloch)
–	Fronto-orbital bristles 1+1 in both sexes	*Tainanina pilisquama* (Senior-White)

#### Key to the species of *Polleniopsis*

**Table:** 

1	Presutural *ia* present, abdomen black, densely covered with yellowish gray or golden dusting, strongly tessellated; facial carina well developed; outer *ph* present	*Polleniopsis toxopei* (Senior-White)
–	Presutural *ia* absent, abdomen black, with bluish or bronzy tinge, silver-gray dusted; facial carina poorly developed; outer *ph* absent	*Polleniopsis dalatensis* Kurahashi

### Subfamily MELANOMYINAE

#### Key to the genera of MELANOMYINAE

**Table:** 

1	Eye bare; *ia* 0 (male) or 1 (female) +2; outer *ph* present; *ac* 2+3; *dc* 3+3; sternopleural bristle (*st*) 1+1; body yellowish brown, thorax with gray dusting; pronotum with 5 black vertical stripes; meso- and metathoracic spiracle yellowish white; leg yellowish, with blackish tarsus; 6.5–7.5 mm	*Tricycleopsis* Villeneuve, *Tricycleopsis paradoxa* Villeneuve
–	Eye hairy, or with sparse short hairs, or bare; *st* usually 2+1; other characters not as above	2
2	Eye with distinct, dense hairs; body almost all black	*Paradichosia* Senior-White
–	Eye with sparse short hairs or bare	3
3	At least part of body yellow; scutellum with at least apex yellowish; T_1+2_ and T_3_ testaceous yellow; legs testaceous except for fuscous tarsi	*Gymnadichosia* Villeneuve, *Gymnadichosia pusilla* Villeneuve
–	Body blackish; scutellum entirely black; abdomen and legs black except for brownish tibia	*Pollenomyia* Séguy, *Pollenomyia sinensis* Séguy

#### Key to the species of *Paradichosia*

**Table:** 

1	Scutellum entirely black	2
–	Scutellum with at least apex yellowish	3
2	Hind tibia with submedian anterodorsal bristle (*ad*) more than 3 times as long as tibial diameter; hind tarsus with dorsal hairs longer than usual, forming a short fringe; third sternite (ST_3_) of male with tuft of long hairs	*Paradichosia dubia* Malloch [not recorded from Taiwan]
–	Submedian *ad* of hind tibia no more than 3 times as long as tibial diameter; dorsal hairs of hind tarsus not longer than usual; male ST_3_ with tuft of very short hairs	*Paradichosia lui* sp. n.
3	Fore coxa yellow in front; femora entirely fulvous yellow; none of the hind tibial bristles exceptionally long or slender in both sexes	*Paradichosia crinitarsis* Villeneuve
–	Fore coxa fuscous in front; femora fulvous yellow or dark brown; 1 or 2 of the hind tibial bristles in male 2–3 times as long as tibial diameter	*Paradichosia nigricans* Villeneuve

### Subfamily LUCILIINAE

#### Key to the genera of LUCILIINAE

**Table:** 

1	Supraspiracular convexity bare or pubescent	*Lucilia* Robineau-Desvoidy
–	Supraspiracular convexity clothed with long, erect, fine hairs	*Hemipyrellia* Townsend, *Hemipyrellia ligurriens* (Wiedemann)

#### Key to the species of *Lucilia*

**Table:** 

1	Postsutural *ac* 3–4	2
–	Postsutural *ac* 2	5
2	Basicosta yellow	3
–	Basicosta fuscous to black	4
3	Abdomen usually metallic green, more or less oval, not arched in profile, without tuft of long hairs on sternites in male; female having metallic green to gold tinged abdomen with sparse pruinosity; 5–8 occipital hairs (*occ*) in female	*Lucilia sericata* (Meigen)
–	Abdomen coppery, elongate, arched in profile, with tufts of long hairs on sternites in male; hypopygium prominent; female having coppery abdomen with dense pruinosity; 1 *occ* in female	*Lucilia cuprina* (Wiedemann)
4	Body metallic purple or blue	*Lucilia taiwanica* Kurahashi & Kano
–	Body metallic green	*Lucilia hainanensis* Fan
5	Anterior pair of postsutural *ac* usually more advanced than second pair of postsutural *dc*; T_3_–T_5_ without dark marginal band posteriorly; mid tibia with 1 *ad* in both sexes	*Lucilia porphyrina* (Walker)
–	Anterior pair of postsutural *ac* level with or slightly posterior to second pair of postsutural *dc*; T_3_–T_5_ with dark marginal band posteriorly; mid tibia with 1 *ad* in male, 2 *ad* in female	6
6	First postsutural *ac* more posterior, on posterior 2/5 part of postsutural area; distance between first postsutural *dc* and second postsutural *dc* twice as long as distance between second postsutural *dc* and third postsutural *dc*; large size flies, body length usually more than 10.0 mm	*Lucilia sinensis* Aubertin
–	First postsutural *ac* normal, on anterior 3/5 part of postsutural area; distance between first postsutural *dc* and second postsutural *dc* as long as distance between second postsutural *dc* and third postsutural *dc*	7
7	Alar squama (upper calypter) creamy, with tuft of yellowish white hairs at inner lower margin; thoracic squama pale, brownish on disc	*Lucilia bazini* Séguy
–	Alar squama fuscous brown, sometimes more or less paler at inner 1/2, but usually with tuft of blackish-brown, sometimes brown, hairs at inner lower margin	8
8	Posterior surface of postgena clothed with yellow hairs; parafacilia narrow, invisible in profile, about 1/2–2/3 of the width of AS_3_, golden yellow dusted, often darkened above	*Lucilia calviceps* Bezzi
–	Posterior surface of postgena clothed with black hairs; parafacilia rather broad, at least as broad as the width of AS_3_, gray dusted, rarely with yellow tinge	*Lucilia papuensis* Macquart

### Subfamily BENGALIINAE

#### Key to the species of *Bengalia*

**Table:** 

1	Prealar knob pointed; fourth sternite (ST_4_) with 1 pair of long and strong median marginal bristles (*mb*) in male; abdomen very slightly pollinose; body small- to medium-sized; mesonotum blackish on disc	*Bengalia calilungae* Rueda
–	Prealar knob rounded; ST_4_ without strong *mb* in male; abdomen slightly to heavily tessellated; body medium- to large-sized	2
2	Fifth tergite without discal bristles; hind tibia never fringed in male	3
–	Fifth tergite with 1 pair of discal bristles; hind tibia more or less fringed in male, sometimes not very dense, covered with row of 3–7 fine long anteroventral bristles (*av*) on apical 1/2	6
3	Vibrissa far above oral margin, distance between vibrissa and oral margin larger than wide of AS_3_; clypeus strongly projecting forward; posterior margin of eye somewhat concave at middle; fore tibia in male with 5+2 ventral spines; hairs on pteropleuron wholly yellowish (Fig. 1)	*Bengalia torosa* (Wiedemann)
–	Vibrissa level with oral margin; clypeus less strongly projecting; posterior margin of eye straight, not concave at middle; fore tibia in male with 3 ventral spines; hairs on pteropleuron wholly whitish yellow or with some black hairs present at least on upper part	4
4	Epaulet (tegula) blackish, with hind margin yellowish; pteropleuron hairs totally whitish yellow (Fig. 2)	*Bengalia chekiangensis* Fan
–	Epaulet yellowish; pteropleuron hairs not totally yellowish	5
5	Fifth tergite black, tessallated; mesopleuron bicolored, yellow on upper 1/3, yellow upper part distinct from lower 2/3 blackish; pteropleuron largely covered with black hairs (Fig. 3); stenopleuron also mostly blackish haired; femora largely fuscous	*Bengalia escheri* Bezzi
–	Fifth tergite yellowish brown with black marginal band; mesopleuron yellowish, but more or less brownish on lower part; pteropleuron largely covered with yellow hairs except for tuft of several blackish hairs (Fig. 4); sternoplerual hairs yellow except for a tuft of black ones; femora yellow	*Bengalia fuscipennis* Bezzi
6	Pteropleuron mostly clothed in yellow hairs, less than 15 black hairs present on upper part (Fig. 5); *dc* 2+4; mid tibia not typically fringed in male; fore tibia with 2–3 long and 2–5 short stout spines on anteroventral surface in male; body 8–11 mm	*Bengalia varicolor* (Fabricius)
–	Pteropleuron mostly or largely clothed in black hairs, at least 20 hairs on upper part black (Fig. 6); *dc* 1–3+4; mid tibia with or without fringe in male; fore tibia without spines, at most with several small spines on anteroventral surface; body 11–15 mm	7
7	Projection of male ST_5_ rounded with small indentation; mid tibia double-fringed on apical 2/3 of posteroventral surface in male; T_5_ in female without indentation in median part of posterior margin; *dc* 3+4	*Bengalia emarginata* Malloch
–	Fifth sternite in male with two–branched projection; mid tibia not typically fringed in male; T_5_ in female with small indentation in median part of posterior margin; *dc* 1–2+4	*Bengalia taksina* (Lehrer)

**Figure 1.**
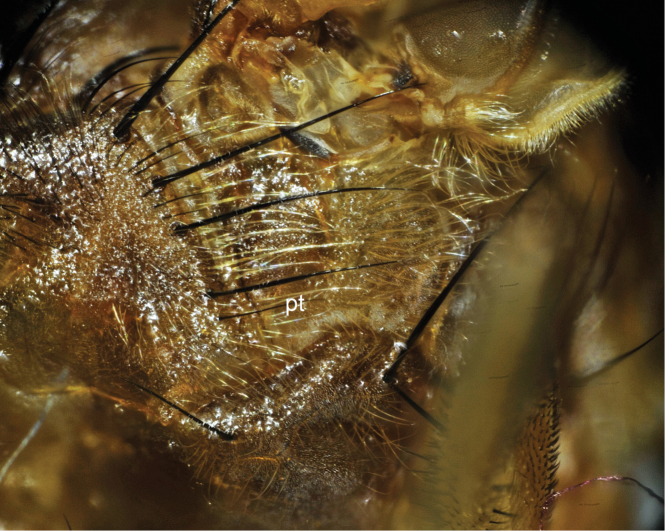
Pteropleuron (pt) of *Bengalia torosa* (Wiedemann), hairs on pteropleuron are wholly yellowish.

**Figure 2.**
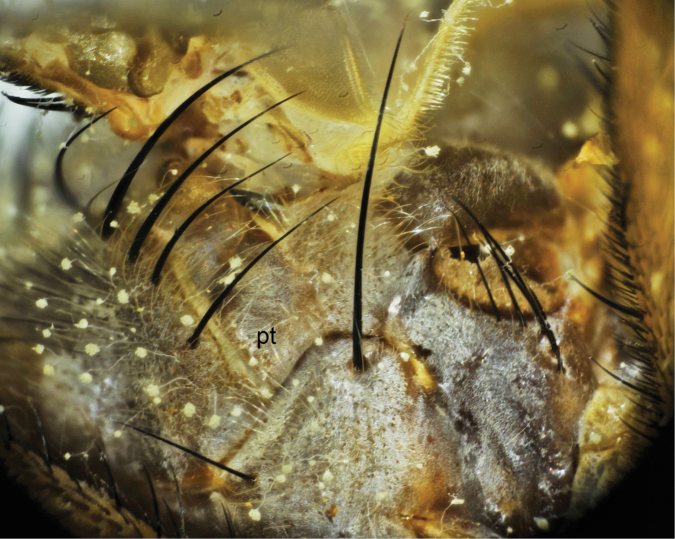
Pteropleuron (pt) of *Bengalia chekiangensis* Fan, hairs on pteropleuron are totally whitish yellow.

**Figure 3.**
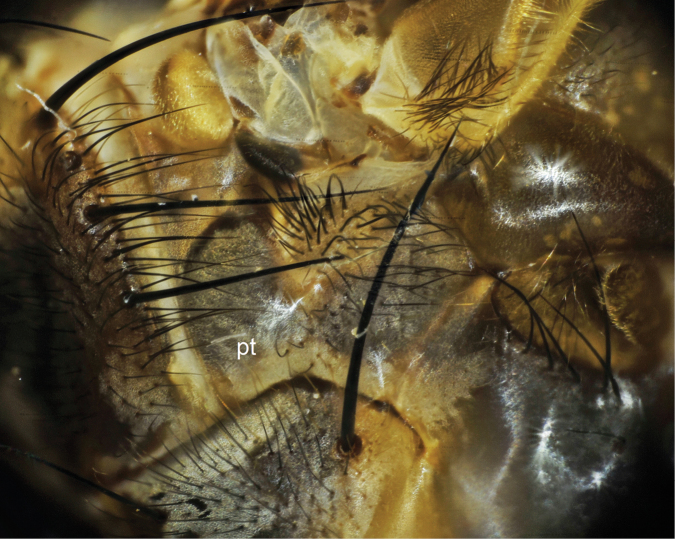
Pteropleuron (pt) of *Bengalia escheri* Bezzi, hairs on pteropleuron are largely blackish.

**Figure 4.**
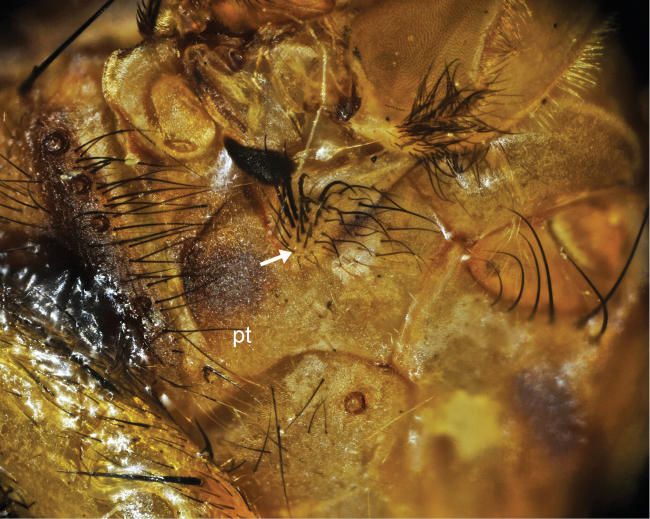
Pteropleuron (pt) of *Bengalia fuscipennis* Bezzi, which is largely covered with yellow hairs except for a tuft of blackish hairs. Arrow shows the blackish hair tuft.

**Figure 5.**
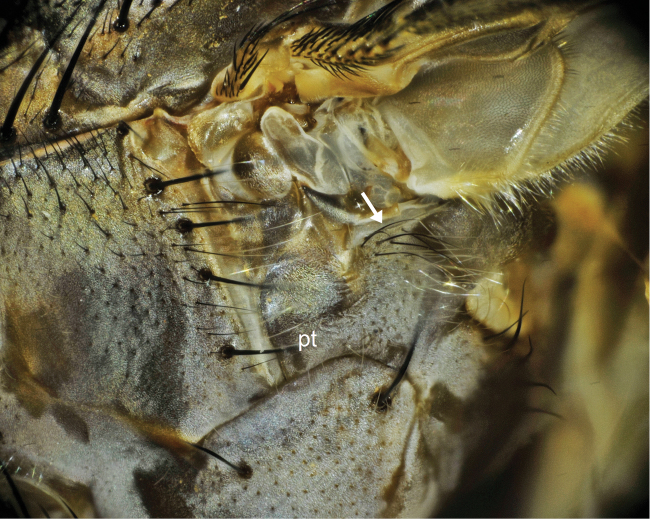
Pteropleuron (pt) of *Bengalia varicolor* (Fabricius), which is mostly clothed in yellow hairs, with a few black hairs present on upper part. Arrow shows the blackish hairs.

**Figure 6.**
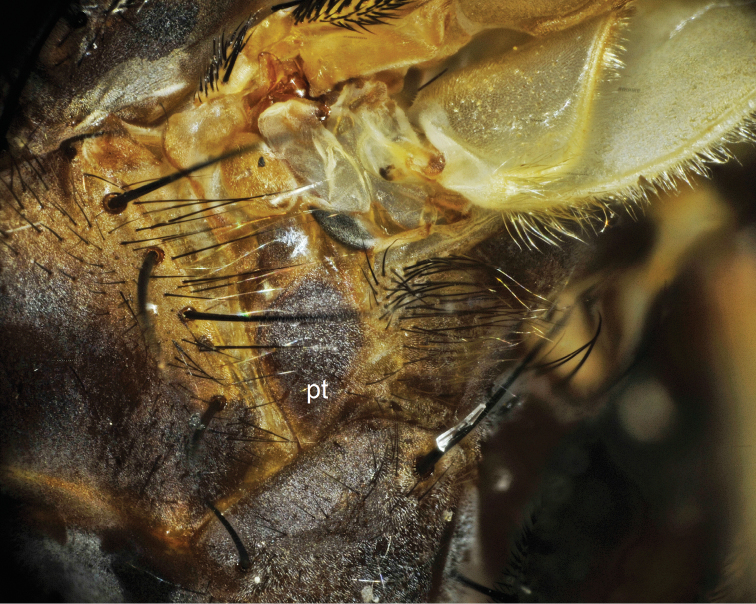
Pteropleuron (pt) of *Bengalia emarginata* Malloch, which is mostly clothed in black hairs.

### Subfamily PHUMOSIINAE

#### Key to the species of *Caiusa*†

**Table:** 

1	Mesonotum with fuscous median stripe or spots; male cercus much shorter than surstylus	*Caiusa* sp. ‡
–	Mesonotum largely fuscous black; male cercus as long as surstylus	*Caiusa indica* Surcouf

† Species *Caiusa testacea* Senior-White is excluded from this key, since the morphological characters that used to recognize *Caiusa testacea* (e.g., mesonotum all pale testaceous yellow) is not autapomorphies of this species (Rognes, personal communication), and we did not have Taiwanese *Caiusa testacea* specimens to examine for building a new couplet.

‡ The taxonomic status of this species is not clear at present. Its external morphology is similar to that of *Caiusa coomani* Séguy (Rognes 2011b), while the hypopygium and genitalia are quite different. It might be an undescribed species or only a variation in *Caiusa testacea* Senior-White (Rognes, personal communication).

### Subfamily POLLENIINAE

#### Key to the genera of POLLENIINAE

**Table:** 

1	Parafacial hairy; body mainly black	*Pollenia* Robineau-Desvoidy [not recorded from Taiwan]
–	Parafacial bare; body entirely or largely testaceous yellow, rarely entirely black	*Dexopollenia* Townsend

#### Key to the species of *Dexopollenia*

**Table:** 

1	Presutural *ac* absent	*Dexopollenia luteola* Villeneuve
–	Presutural *ac* present	2
2	Thorax yellow to dark brown; abdomen entirely yellow, sometimes with black spots on T_4_ and T_5_; leg almost entirely yellow except for fuscous tarsus; AS_3_ largely yellow	*Dexopollenia flava* (Aldrich)
–	Thorax entirely black; abdomen with blackish median stripe; male femora and tibia entirely fuscous black, and entirely orange in female; AS_3_ entirely brown	*Dexopollenia maculata* Villeneuve

### Subfamily CHRYSOMYINAE

#### Key to the tribes of CHRYSOMYINAE

**Table:** 

1	Prealar knob with erect hairs	Tribe Chrysomyini
–	Prealar knob without erect hairs	Tribe Phormiini, *Protocalliphora* Hough

#### Key to the genera of Chrysomyini

**Table:** 

1	Sternopleural bristle (*st*) 0+1; head dichoptic in both sexes	*Ceylonomyia* Fan, *Ceylonomyia nigripes* (Aubertin)
–	Sternopleural bristles 1+1; head holoptic to dichoptic in male, dichoptic in female	2
2	Outer vertical bristle (*ov*) developed in both sexes; no proclinate *ors* developed in female (*ors* 0+1); T_5_ in female with cleft on median part of posterior margin [larvae facultative predacious species, body with fleshy tubercles]	*Achoetandrus* Bezzi
–	Outer vertical bristle absent in male, developed in female; 2 proclinate *ors* (*ors* 2+1) developed in female; T_5_ in female without median cleft, truncate posteriorly	*Chrysomya* Robineau-Desvoidy

#### Key to the species of *Achoetandrus*

**Table:** 

1	Mesothoracic spiracle white or yellow; femora normal in both sexes; head holoptic in male, dichoptic in female; facial ridge not remarkably high; T5 with some pale hairs laterally among black ones	*Achoetandrus rufifacies* (Macquart)
–	Mesothoracic spiracle fuscous black to dark brown; femora swollen in both sexes, more noticeably so in male; head dichoptic in both sexes; facial ridge well developed, high	*Achoetandrus villeneuvi* (Patton)

#### Key to the species of *Chrysomya*

**Table:** 

1	Gena and postgenal area entirely orange-yellow, clothed with pale yellow hairs, except immediately around vibrissa	2
–	Gena and postgenal area entirely fuscous or somewhat rufous anteriorly, entirely clothed in black hairs or with some pale hairs posteriorly	*Chrysomya pinguis* (Walker)
2	Alar and thoracic squama entirely white except for yellowing of fringe; mesothoracic spiracle small, not longer than length of AS_3_; upper eye facets not conspicuously larger than lower in male; parafrontal not obliterated in male; eyes separated by slightly less than width of AS_3_ in male [myiasis-producing species rarely found except by rearing from hosts]	*Chrysomya bezziana* Villeneuve
–	At least thoracic squama distinctly infuscated posteriorly; mesothoracic spiracle large, much longer and broader than AS_3_ in profile; male with upper eye facets conspicuously enlarged and with sharp transition to small facets in lower 1/3; parafrontal almost obliterated and eyes virtually touching above in male [common synanthropic species]	*Chrysomya megacephala* (Fabricius)

### Subfamily RHINIINAE

#### Key to the tribes and genera of RHINIINAE

**Table:** 

1	Arista pectinate; *ac* and *dc* not distinguishable from general vestiture, prescutellar bristles at most weakly developed; suprasquamal ridge bare	Tribe Rhiniini, 2
–	Arista pubescent or plumose, not pectinate	Tribe Cosminini, 5
2	Outer *ph* absent; cell R_5_ petiolate; male head holoptic, female dichoptic; body metallic green or dark blue	*Chlororhinia* Townsend [not recorded from Taiwan]
–	Outer *ph* present; cell R_5_ variable; male head variable, female dichoptic; body variable in color	3
3	Hind tibia without conspicuous row of *ad*, but with 2–3 *ad* as long as or longer than tibial diameter; cell R_5_ open; body slender, parallel-sided; abdomen mostly testaceous	*Idiella* Brauer & Bergenstamm
–	Hind tibia with conspicuous row of subequal setae on anterodorsal surface, longer than general vestiture, sometimes 2–3 rather strong *ad* developed among them; fore tibia with 1 *pv*; body rather stout with ovate abdomen, usually of dark coloration	4
4	Cell R_5_ petiolate; stenopleuron brassy, without pollinosity; mesopleuron without setigerous spots; legs entirely yellow	*Rhinia* Robineau-Desvoidy
–	Cell R_5_ open at wing margin or closed; if petiolate then sternopleuron heavily dusted	*Stomorhina* Rondani
5	Arista plumose, rays at least as long as width of AS_3_ in anterior view; fore tibia without or with 1 posterior bristle (*p*)	6
–	Arista pubescent, longest hairs never exceeding 1.5 times width of AS_3_ in anterior view; fore tibia with 1 *p*	*Rhyncomya* Robineau-Desvoidy
6	Outer *ph* absent; fore tibia without *p*	7
–	Outer *ph* present; fore tibia with 1 *p*, rarely lack in *Malayomyza*	8
7	Prostigmatal bristles (*pst*) absent; two longitudinal silver white stripes present on dorsum	*Borbororhinia* Townsend, *Borbororhinia bivittata* (Walker)
–	Prostigmatal bristles present; yellowish brown dorsum with or without three fuscous longitudinal stripes, or fuscous dorsum with two longitudinal silver white to grey stripes	*Sumatria* Malloch
8	Presutural *ac* absent or indistinct; *dc* and postsutural *ac* usually indistinct except for prescutellars; if 1–2 postsutural *ac* developed as prescutellars, then propleuron hairy	*Cosmina* Robineau-Desvoidy [not recorded from Taiwan]
–	Presutural *ac* well developed at least in one pair; *dc* also well developed; propleuron bare	9
9	Fore tibia without *p*; small fly, less than 4 mm in length, blackish and shiny, with bronzy tinge; humerus, propleuron, upper and anterior part of mesopleuron reddish brown; abdomen reddish brown in part on T_1+2_ and T_3_; male head dichoptic	*Malayomyza* Malloch [not recorded from Taiwan]
–	Fore tibia with 1 *p*; medium- to large-sized fly having thorax usually metallic green, blue and purple, more or less pollinose; humerus, propleuron, and mesopleuron concolorous with thoracic dorsum; abdomen concolorous with thoracic dorsum, also pollinose, sometimes tessellated; male head usually holoptic to subholoptic	10
10	Mesopleuron with group of bristles on its upper part; bend of vein M_1+2_ regularly curved or angulose; male hypopygium of normal size, scarcely visible on abdomen in profile; female last sternite not projecting posteriorly	*Isomyia* Walker
–	Mesopleuron without bristle on its upper part next to first notopleural bristle; bend of vein M_1+2_ gently curved; male hypopygium and ST_5_ very strongly developed, altogether almost same size as rest of abdomen; female last sternite widely uncovered by corresponding tergites, its posterior border projecting outwards	*Strongyloneura* Bigot

#### Tribe Rhiniini

##### Key to the species of *Stomorhina*

**Table:** 

1	Mesopleuron with 1–2 bristles on upper corner of posterior margin	2
–	Mesopleuron with complete row of black bristles along posterior margin; wing strongly infuscated along costal border and with distinct apical suffusion; fore tibia blackish	*Stomorhina veterana* Villeneuve
2	Mesopleuron without distinct piliferous spots	3
–	Mesopleuron with distinct piliferous spots	5
3	Sternopleuron as densely yellow pollinose as mesopleuron	4
–	Sternopleuron and hypopleuron glossy black, not pollinose; cell R_5_ open; abdomen blackish, with violet tinge	*Stomorhina melastoma* (Wiedemann) [not recorded from Taiwan]
4	Thoracic squama with lobulate inner border; cell R_5_ closed, petiolate	*Stomorhina xanthogaster* (Wiedemann)
–	Thoracic squama without lobulate inner border; cell R_5_ open	*Stomorhina lunata* (Fabricius)
5	Hind femur yellowish at base; cell R_5_ open narrowly; anterior lower part of mesopleura and anterior part of sternopleuron glossy, black; male abdomen not pollinose, without piliferous spots; T_1+2_ with only narrow fuscous posterior marginal band, without median stripe	*Stomorhina discolor* (Fabricius)
–	Hind femur entirely dark brown; cell R_5_ open; anterior lower part of mesopleuron and anterior part of sternopleuron black, weakly gray pollinose, not glossy; male abdomen with piliferous spots; T_1+2_ with both fuscous anterior and posterior marginal band, with median stripe	*Stomorhina obsoleta* (Wiedemann)

##### Key to the species of *Idiella*

**Table:** 

1	Basicosta black; occipital dilatation, mesopleuron and sternopleuron with distinct piliferous spots	2
–	Basicosta brown; occipital dilatation, mesopleuron and sternopleuron without distinct piliferous spots	3
2	First visible tergite without lateral bristle among fine yellow general vestiture; hind tibia in male with fine long hairs on posteroventral surface, length of hairs more than tibial diameter	*Idiella divisa* (Walker)
–	First visible tergite with 1 to several black lateral bristles among fine yellow general vestiture; hind tibia in male without fine long hairs on posteroventral surface, tibial hairs not exceeding tibial diameter	*Idiella euidielloides* Senior-White
3	Second antennal segment reddish; mid tibia with 2 *p* and brush of hairs in male; male frons usually broader than width of ocellar triangle	*Idiella mandarina* (Wiedemann)
–	Second antennal segment fuscous; mid tibia with 1 *p*, but without brush of hairs on inner surface of apex in male; male frons variable in width	*Idiella tripartita* (Bigot) [not recorded from Taiwan]

#### Tribe Cosminini

##### Key to the species of *Isomyia*

**Table:** 

1	Thoracic squama strongly lobulate; body very stout	2
–	Thoracic squama generally not lobulated, not reaching base of scutellum, its longitudinal diameter longer than transverse; body usually slender; antenna yellow; *ac* 2+4; alar and thoracic squama yellowish white	*Isomyia tibialis* (Villeneuve)
2	Mesopleural hairs and hairs of other pleural areas as well, soft and yellow to golden, except for usual black setulae just below notopleural suture; mesothoracic spiracle yellow	3
–	Mesopleural hairs more extensively black than indicated above, with some soft black hairs on mesopleuron, sometimes remote from notopleural suture, and on sternopleuron	4
3	Basicosta bright yellow; epaulet yellowish; pleura and abdomen densely pollinose in male, less so in female, but dorsum of T_5_, when viewed laterally at angle, with tessellated pattern of pollinosity; black lateral bristles on T_1+2_ surrounded, at least on three sides, by pale yellow hairs	*Isomyia viridaurea* (Wiedemann)
–	Basicosta black; epaulet black; T_3_ and T_4_ without marginal band; wing hyaline, sometimes slightly infuscated apically in female; parafrontal in female subequal to frontal stripe at middle of frons; hind tibia without *av* in male	*Isomyia electa* (Villeneuve)
4	Marginal scutellar bristles (*msc*) 4, the last three spaced at closer intervals than basal bristle; both alar and thoracic squama pale yellow; posterior mesopleural fringe yellow; some hairs on notopleuron, lower part of mesopleuron, and part of sternopleuron yellow to yellowish brown	*Isomyia pseudolucilia* (Malloch)
–	Marginal scutellar bristles 3, spaced at approximately equal intervals	5
5	Pleura with extensive yellow hairs, at least around *pst* and propleural bristles (*pp*) and on part of sternopleuron; posterior mesopleural fringe golden	6
–	Pleural hairs entirely black or virtually so; posterior mesopleural fringe brown to black; alar and thoracic squama wholly dark brown to black	*Isomyia oestracea* (Séguy)
6	Mesothoracic spiracle entirely bright yellow to golden; T_3_ and T_4_ without marginal band; wing hyaline, sometimes slightly infuscated apically in female; parafrontal in female subequal to frontal stripe at middle of frons; hind tibia without *av* in male	*Isomyia electa* (Villeneuve)
–	Mesothoracic spiracle dark brown to black	*Isomyia delectans* (Walker)

##### Key to the species of *Rhinia*

**Table:** 

1	Mesopleura, and usually occipital dilatation too, with distinct piliferous spots; sternopleura pruinose; cell R_5_ open	*Rhinia sauteri* Peris
–	Mesopleura without distinct piliferous spots; male scutellum with long and fine hair; interfrontalia width twice each parafrontal in female	*Rhinia apicalis* (Wiedemann)

##### Key to the species of *Rhyncomya*

**Table:** 

1	Propleuron hairy; T_3_ and T_4_ with black median stripe; T_4_ with dark marginal band; T_5_ blackish	*Rhyncomya notata* (van Der Wulp)
–	Propleuron bare; antenna yellowish fuscous, AS_3_ brownish; posterior mesopleural bristles 6–7; hind tibia with 2 fine *av.* Female frons as wide as 0.67 eye wide; interfrontal area reddish brown, not wider at lunule; femur entirely black	*Rhyncomya setipyga* Villeneuve

##### Key to the species of *Strongyloneura*

**Table:** 

1	Male sternite without brush-like tuft of hairs or bristles except ST_5_; anteroventral side of hind coxa with yellow fine hairs and black hairs	*Strongyloneura prasina* Bigot
–	Male with brush-like tuft of hairs or bristles on each sternite	2
2	Third sternite with large brush-like tuft of hair	*Strongyloneura diploura* Fang & Fan
–	Third sternite without tuft of hair; ST_4_ with tuft of hair	*Strongyloneura prolata* (Walker)

##### Key to the species of *Sumatria*

**Table:** 

1	Dorsum of thorax orange, with paired narrow longitudinal brownish stripes separated by a median silvery gray-dusted area	*Sumatria flava* (Villeneuve)
–	Dorsum of thorax testaceous, with three dark longitudinal stripes	2
2	Dorsum of thorax with three dark longitudinal stripes separated by silvery gray-dusted areas	*Sumatria chiekoae* Kurahashi & Tumrasvin
–	Dorsum of thorax with three dark longitudinal stripes, without gray pruinosity	*Sumatria vittata* (Peris)

##### New taxon

###### Paradichosia lui sp. n.

Taxon classificationAnimaliaDipteraCalliphoridae

http://zoobank.org/23029B64-E9A8-4A44-B292-E88E08201588

Figs 7 , 9

####### Description.

Male. Head: Holoptic, eyes hairy; frons index < 0.01; frontal stripe dark brown to black, obliterated at narrowest point of frons; parafrontal dark brown to black, silver pollinose, setulose, with about 7 pairs of orbital bristles (*ori*); parafacial dark brown to black, silver pollinose, setulose, about as wide as width of AS3; face dark brown to fuscous, slightly gray pollinose, facial carina not developed; facialia dark brown to fuscous, gray pollinose, with several hairs above vibrissa; mediana dark brown, slightly pollinose, with some hairs below; vibrissaria brown, with several bristles; vibrissa developed; epistome yellowish, slightly projecting forward, not obviously demarcated from face; gena blackish, gray pollinose, clothed with black hairs; postgena concolorous with gena, not demarcated from gena, with mostly black hairs but yellowish hairs posteriorly; occiput concolorous with gena, whitish gray-dusted, clothed in yellowish soft hairs at inner part and black hairs along outer margin, epicephalon and upper parts along posterior eye margins black, slightly dusted; AS_2_ fuscous, reddish to brown, at joint of AS_3_ pale apically; AS_3_ fuscous, somewhat reddish to brown at joint of AS_2_, yellowish white pollinose, about 3 times as long as AS_2_; arista dark brown, long plumose on basal 2/3; palpus orange, with black setulae.

Thorax: blackish, thinly whitish gray pollinose on dorsum and pleura; humerus, postalar callus and scutellum concolorous with thoracic dorsum; prosternum black, with brown hairs below; propleuron yellowish to blackish, whitish pollinose posteriorly, with yellowish brown hairs; mesopleuron and sternopleuron clothed with black hairs and bristles; supraspiracular convexity pubescent, without long upstanding hairs; hypopleuron clothed in black hairs and bristles; other pleural hairs also blackish; mesothoracic spiracle blackish brown on upper part, lower part brown; metathoracic spiracle fuscous; postalar declivity fuscous black, with black hairs; tympanic tuft present; anterior parasquamal tuft present.

Chaetotaxy: *ac* 2+3; *dc* 2+3; *ia* 1+2; humeral bristles (*h*) 3; *ph* 2-3, 2 strong, 1 fine bristle present in front of strong inner *ph*; *prs* 1; supraalar bristles (*sa*) 3; postalar bristles (*pa*) 3; *st* 2+1; *msc* 5; discal scutellar bristle (*dsc*) 1; notopleural bristles (*n*) 2; propleural bristles (*pp*) more than 5; *pst* 1.

Wings: Brownish hyaline; veins brown; epaulet black, with black setulae and bristles; basicosta blackish; subcostal sclerite dark brown, yellowish pubescent; node of 2nd (R_2+3_) and 3rd (R_4+5_) longitudinal veins with a few black setulae above and below; 4th longitudinal vein (M_1+2_) forming right angle; cell R_5_ open in wing margin; section of 4th vein from bend to wing margin slightly curved inward; alar squama fuscous, paler at base with golden brown pubescence, semitransparent on apical part, with tuft of dark brown hairs at inner lower margin; thoracic one fuscous, lobulate bare on dorsal surface. Halter brownish, with yellowish apex.

Legs: Femora dark brown to black, tibia brownish, tarsi blackish; fore coxa dark brown to black, more or less whitish grey pollinose; mid and hind coxae brownish black, more or less whitish grey pollinose; fore tibia with 1 *p* and row of several short *ad*; mid tibia with 1 *ad*, 1 *p*, 2 *pv*, and 1 *v*; hind tibia with 3 *ad*, 2–3 *pd*, and 2–3 *av.*

Abdomen: Blackish, submetallic, with bronze tinge, slightly whitish grey pollinose, fine black median stripe more or less distinct on T_3_; hairs and bristles black; T_1+2_ with 2–4 lateral marginal bristles; T_3_ with lateral and median *mb*; T_4_ with row of *mb*; T_5_ with row of *mb* and several discal bristles; ST_2_ large, elongated, blackish, whitish gray pollinose except for margin, clothed in black hairs only; ST_3_ and ST_4_ with tuft of short hair as shown in Fig. 9G, H. ST_5_ with lobes narrower and bended inward at tip as shown in Fig. 9C.

Hypopygium small in size, withdrawn from sight; genitalia as shown in Fig. 9A, B, D, E, F, aedeagus with fine and slender paraphallus and acrophallus.

Female. Unknown.

Length: 9.5 mm

Holotype ♂, TAIWAN, Yilan, Datong Township, Jiuliao River, *ca.* 158 m, riverside, 26.i.2013, S. T. Yang (NTU). Paratypes, 1♂, Tatung, 28.xii.1991, C. L. Chung (NSMT); 1♂, Tatung, Chilanshan, Chihtuan, 15.xi.1961, J. C. Lien (NSMT).

**Figure 7.**
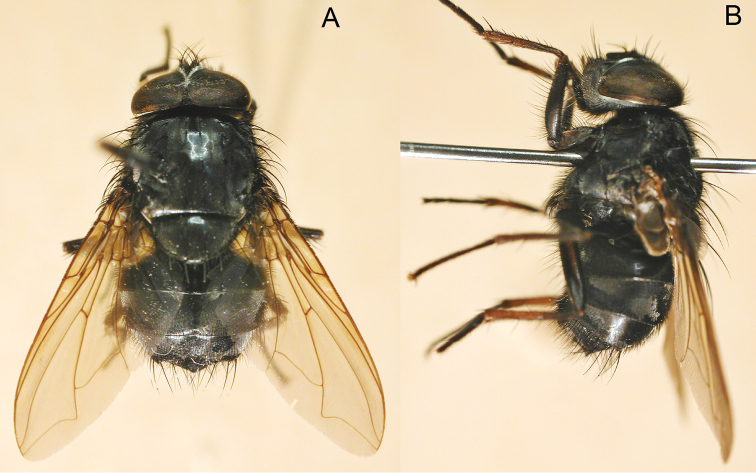
*Paradichosia lui* sp. n., ♂. **A** dorsal view **B** lateral view.

####### Etymology.

The specific epithet *lui* is named after Mr. I-Tse Lu, who guided the first author to the collecting site and helped collect specimens.

####### Type depository.

Holotype (♂) is deposited in the Insect Museum of National Taiwan University (NTU); two paratypes (type series NSMT-I-Dip6958, 6959) are deposited in the National Museum of Nature and Science, Tokyo (NSMT).

####### Diagnosis.

This new species, which has hairy eyes and a blackish scutellum, is similar to *Paradichosia dubia* (Malloch) from Java, Indonesia in general morphology. Nevertheless, it can be easily distinguished from *Paradichosia dubia* by having very short hairs on the tuft of ST_3_ in the male.

####### Bionomics.

Adults were found on the bush near the stream side.

####### Distribution.

Taiwan (Yilan).

##### New Record from Taiwan

###### Silbomyia hoeneana

Taxon classificationAnimaliaDipteraCalliphoridae

Enderlein

Figs 8 , 10

####### Redescription.

Male. Head: Eyes dichoptic; frons index 0.42; frontal stripe yellow to yellowish-orange, very wide, about 4 times as wide as a parafrontal, black setulose on outer part; parafrontal yellow, silvery-yellow pollinose, with about 9 pairs of *ori*, reclinate *ors* 1, proclinate *ors* 2; parafacial yellow, silvery-yellow pollinose, bare, about 2 times as wide as width of AS_3_; face yellow, slightly yellowish pollinose; facial carina developed, rather short and broad, shorter than distance from lunule to median ocellus, more or less ridge-like, not fusiform, facialia yellow, slightly yellowish pollinose, with several black hairs above vibrissa; mediana yellow, silvery-yellow pollinose; vibrissaria yellow, with several strong bristles; vibrissa well developed; epistome yellow, not obviously demarcated from face; gena yellow, silvery-yellow pollinose, covered with yellowish-fuscous to brown hairs; peristomal bristles black and stout; postgena concolorous with gena, not demarcated from gena, with yellowish hairs; postorbits dark brown, bright silver pollinose; upper occiput dark brown to black, slightly silvery-gray pollinose, with black hairs, lower occiput yellow, silvery-yellow pollinose, with golden-yellow hairs; upper epicephalon yellow, concolorous with frontal stripe, lower part brown; AS_2_ yellowish to pale orange; AS_3_ yellowish to pale orange, yellowish white pollinose, about 3 times as long as AS_2_; arista fuscous brown, almost entirely long plumose; palpus pale orange, with black setulae.

Thorax: Metallic bluish-green to blue, with somewhat purplish tinge, slightly silvery-gray pollinose on dorsum and pleura; humerus, postalar callus, and scutellum concolorous with thoracic dorsum; outer prosternum yellowish, inner part dark brown, with rather long black hairs; propleuron dark brown, silvery-gray pollinose, with metallic greenish tinge, covered with brown hairs; supraspiracular convexity brownish pubescent, without long upstanding hairs; hypopleural with row of long black bristles; other pleural hairs black; mesothoracic spiracle dark brown; metathoracic spiracle dark brown; postalar declivity fuscous black, with black hairs; tympanic tuft absent; anterior parasquamal tuft absent.

Chaetotaxy: *ac* 3+4–5; *dc* 3+4; *ia* 1+2–4, sometimes 2 weaker postsutural *ia* present; *h* 4–5; *ph* 3; *prs* 1; *prealar* 1; *sa* 2; *pa* 2; *st* 2+1; *msc* 3–4; *dsc* 2–4; *n* 2; *pp* 2; *pst* 1–3.

Wings: Dark brown infuscate, most strongly brown along veins, paler in cells and to hind margin; veins dark brown; epaulet black, with black setulae and bristles; basicosta black, with brown pubescence; subcostal sclerite dark brown; node of R_2+3_ and R_4+5_ veins with several black setulae extending to R_4+5_ above and below; vein M_1+2_ forming right angle; Cell R_5_ open at wing margin; section of vein M_1+2_ from bend to wing margin slightly curved inward; alar squama whitish, with pale yellow edge and white fringe; thoracic squama whitish, bare on dorsal surface, with dark brown edge and dark brown fringe. Halter fuscous, darkened on apex.

Legs: Femora dark brown to blackish, with metallic violet tinge; tibiae brown; tarsi dark brown; coxa dark brown to blackish, covered with black hairs and bristles anteriorly; fore tibia with row of several strong *ad*, 3 short *pd*, 2–3 strong *p*; mid tibia with row of several strong *ad*, several short *pd*, 2–3 strong *p*, 1 strong *av*; hind tibia with 2 *av*, row of several strong *ad*, 4 long *pd.*

Abdomen: Metallic bluish-green to blue, with somewhat purplish tinge, slightly silvery-gray pollinose; hairs and bristles black; T_1+2_ with 1 lateral marginal bristle; T_3_ with lateral and 1 pair of strong median *mb*, sometimes with 1 or a pair of median discal bristles weakly developed; T_4_–T_5_ with row of *mb*; sternites dark brown with metallic violet tinge, covered with black bristles and hairs. Fifth sternite with long and wide lobes, longer than basal part, as shown in Fig. 10C.

Hypopygium not prominent; genitalia as shown in Fig. 10A, B, D, E, F, paraphallus stout, strongly ossified; hypophallus and acrophallus stout.

Female. Head: Eyes dichoptic; frons index 0.54; frontal stripe yellow to yellowish-orange, broad, about 2.8 times as wide as one parafrontal, black setulose laterally; parafrontal yellow, silvery-yellow pollinose, broader than that of male, with about 9 pairs of *ori*, reclinate *ors* 1, proclinate *ors* 2; parafacial yellow, silvery-yellow pollinose, bare, broader than that of male, about 3 times as broad as width of AS_3_; *ors* 2+1; ocellar bristle (*oc*) 1; postocellar bristles (*pooc*) 3–4; *ov* 1; inner vertical bristle (*iv*) 1; postoccipital bristle (*poc*) 1; occipital bristle (*occ*) 1.

Legs: Fore tibia with row of several strong *ad*, 4 *pd*, 2 strong *p*; mid tibia with row of several strong *ad*, 3 *pd*, 2 strong *p*, 1 strong *av*; hind tibia with 2 *av*, row of several strong *ad*, 4 long *pd.* Otherwise same as for male.

Length: 12.0–17.0 mm.

**Figure 8.**
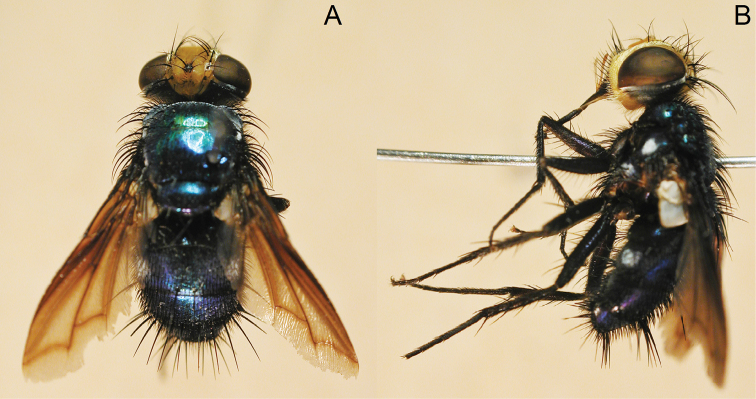
*Silbomyia hoeneana* Enderlein, ♂. **A** dorsal view **B** lateral view.

**Figure 9.**
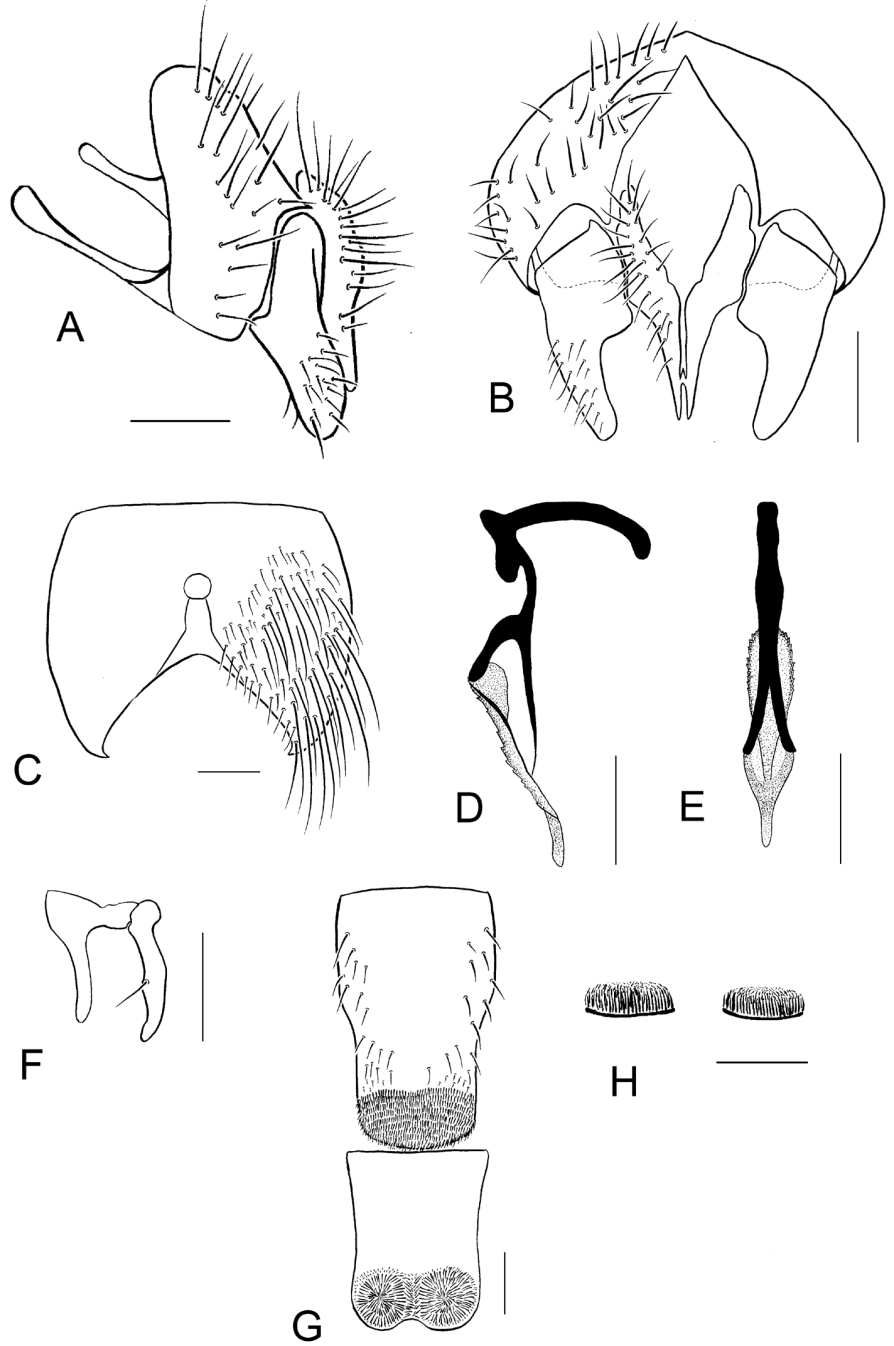
Sternites and genitalia of *Paradichosia lui* sp. n., ♂. **A** Epandrium, cercus, and surstylus, lateral view **B** Cercus and surstylus, caudal view **C** Fifth abdominal sternite, ventral view **D** Aedeagus, lateral view **E** Aedeagus, posterior view **F** Anterior and posterior parameres **G** Third and fourth abdominal sternites, ventral view **H** Tufts on the third (left) and fourth (right) abdominal sternites. Scale bars: 0.2 mm.

**Figure 10.**
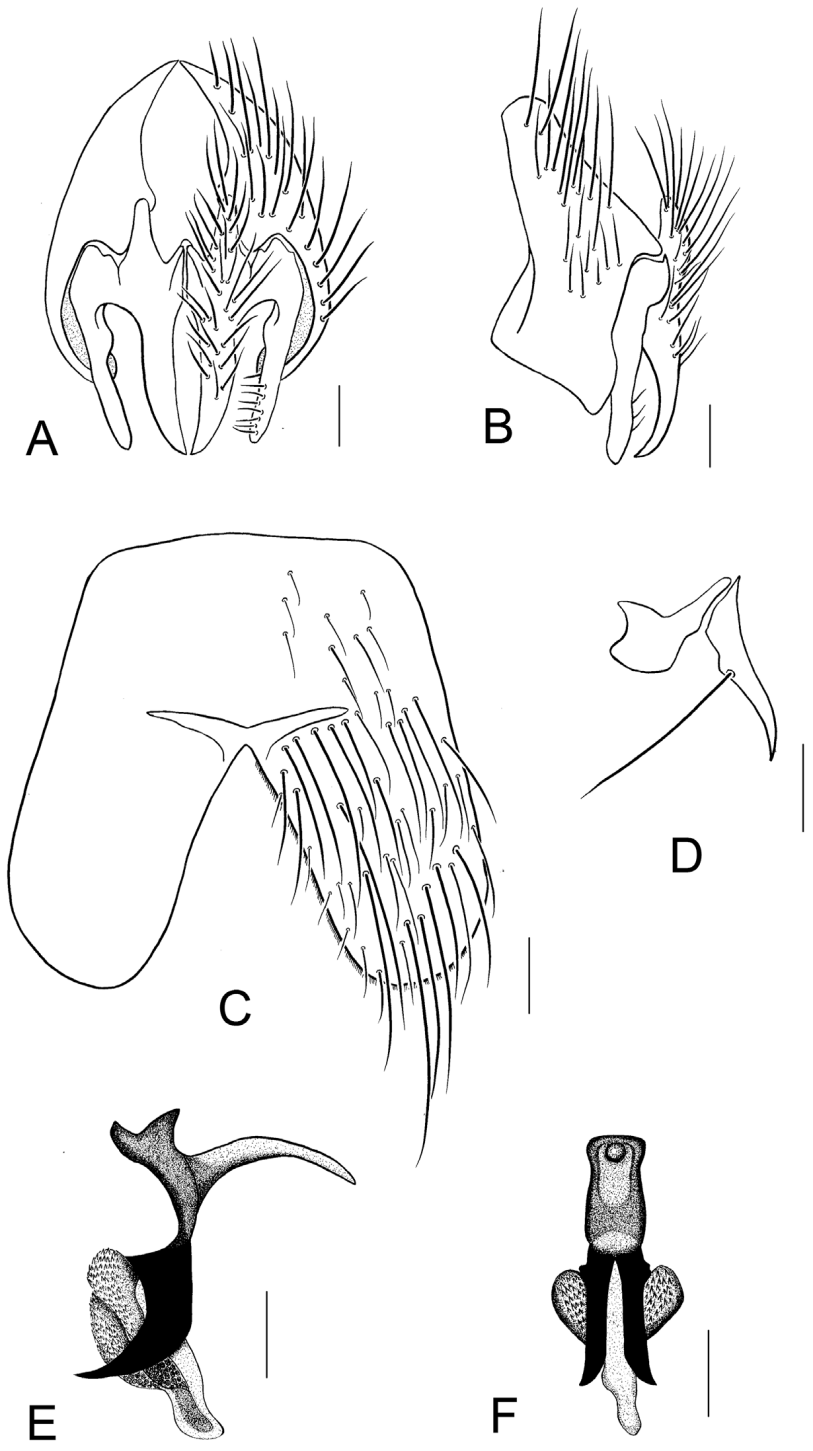
Sternite and genitalia of *Silbomyia hoeneana* Enderlein, ♂. **A** Cercus, and surstylus, caudal view **B** Epandrium, cercus, and surstylus, lateral view **C** Fifth abdominal sternite, ventral view **D** Anterior and posterior parameres **E** Aedeagus, lateral view **F** Aedeagus, posterior view. Scale bars: 0.2 mm.

####### Diagnosis.

**Figure 11.**
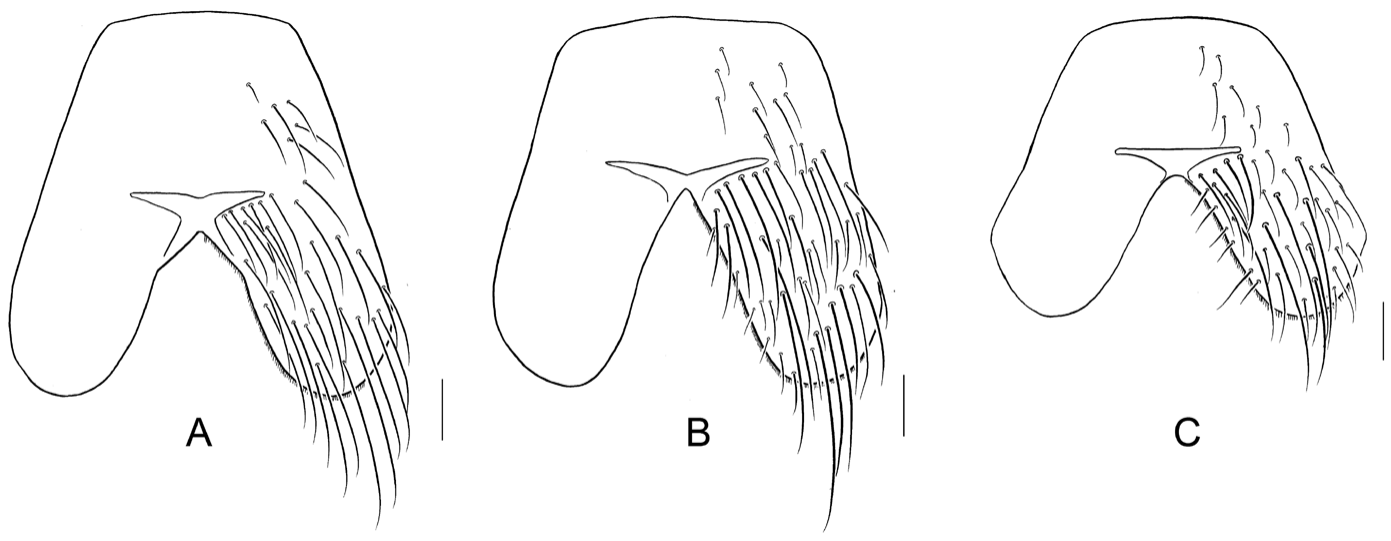
The male fifth abdominal sternites of *Silbomyia* species. **A** *Silbomyia sauteri* **B** *Silbomyia hoeneana*, Taiwan **C** *Silbomyia hoeneana*, China. Scale bars: 0.2 mm.

The specimens of this species collected in Taiwan are slightly different in external morphology from those collected in China. Taiwanese specimens are more bluish when compared to the greener Chinese individuals, and the shape of the ST_5_ is different (Fig. 11) (A, *Silbomyia sauteri*; B, *Silbomyia hoeneana*, collected in Taiwan; C, *Silbomyia hoeneana*, collected in China), the lobes of the ST_5_ of *Silbomyia hoeneana* collected in Taiwan are longer than the basal part, while those of *Silbomyia hoeneana* collected in China are almost equal to the basal part.

####### Bionomics.

Adults are frequent flower visitors.

####### Distribution.

Taiwan and Southern China (Jiangsu Province, Zhejiang Province, Sichuan Province, Jiangxi Province, Hainan Province, Guangdong Province, Yunnan Province).

## Checklist

The following list of Taiwanese Calliphoridae is based mainly on the specimens examined and to a lesser extent on the published records. Names of the collecting localities are based on the original spelling on the collecting labels of specimens.

### Subfamily AMENIINAE

#### Tribe Ameniini

##### *Silbomyia* Macquart, 1843

###### Silbomyia cyanea

Taxon classificationAnimaliaDipteraCalliphoridae

(Matsumura, 1916)

####### Materials.

1♂, Kaohsiung City, Liugui Dist., Tsaidie Valley, 366 m, secondary forest, 31.iii.2013, S. T. Yang (NTU); 1♀, P'ing-tung Hsian, K'en-ting-kung-yuan, 2.iv.1965, R. Kano (NSMT); 1♂, P'ing-tung Hsien, Kuei-chiao-liu, 3.iv.1965, S. Ueno (NSMT); 2♀, T'ai-nan Hsien, Kuan-tzu-ling, 250 m, 6.iv.1965, Rokuro Kano (NSMT); 1♀, Chia-I Hsien, Ch'-hsin-liao, 15.iv.1965, S. Ueno (NSMT); 1♀, Jiji (Chichi), 30.iv.2006, H. Kurahashi (NSMT); 1♀, Kuan-tzu-ling, 250 m, 6.iv.1965, R. Kano (NSMT); 3♀, Puli, 26.ix.1965, K. Kaneko (NSMT); 1♂, Chihpen, 20.vii.1985, S. Shinonaga (NSMT); 1♂, Nanshanchi, 26.vii.1985, S. Shinonaga (NSMT); 1♂, Kenting Park, 15.vii.1985, S. Shinonaga (NSMT); 2♂ 1♀, Chihpen hot spring, 3–4.vi.1992, R. Kano and H. M. Lin (NSMT); 1♂, Kenting, 11.x.1965, K. Kaneko (NSMT); 1♀, Janai (Mushia), 28.ix.1965, K. Kaneko (NSMT); 1♂, Chuchi, 5.x.1965, K. Kaneko (NSMT); 1♂ 1♀, Shanpin, Kaohsiung Co., 6.iv.1996, R. Matsumoto (NSMT); 3♀, Puli, Nanshanhsi, 23-24.v.1992, R. Kano (NSMT); 2♂, Kentin, 15.ii.1972(1♂), 10.iii.1972(1♂), K. Matsuki (NSMT); 2♀, Liuknei, 12.vii.1985, S. Shinonaga (NSMT).

###### Silbomyia sauteri

Taxon classificationAnimaliaDipteraCalliphoridae

Enderlein, 1936

####### Materials.

1♂, Tainan City, Baihe Dist., Guanziling, Red-Leaf Park, 357 m, secondary forest, 21.iii.2013, S. T. Yang (NTU); 2♀, Santimen, 13.vii.1985, S. Shinonaga (NSMT); 2♀, Kentin, 15.ii.1972(1♀), 10.iii.1972(1♀), K. Matsuki (NSMT); 1♀, Taipei Fushan Botanical Garden, 700 m, 24–26.x.2002, M. Owada (NSMT); 1♀, Kuan-tzu-ling, 7.iv.1965, S. Miyamoto (NSMT); 1♀, Jih-yueh-tan, 24.ix.1965, K. Kaneko (NSMT); 1♀, Lushan, 1,000 m, 24–25.vii.1985, S. Shinonaga (NSMT); 2♂, Shannpin, Nantou Co., 7.iv.1996, R. Matsumoto (NSMT); 1♂, Jiji (Chichi), 30.iv.2006, H. Kurahashi (NSMT); 1♀, Fushan B. G., 600 m, 3–6.v.2006, H. Kurahashi (NSMT); 1♀, Kuanzuling, 7.x.1965, K. Kaneko (NSMT); 1♂, Paling, Taoyuen, 9–11.vi.1992, R. Kano (NSMT).

###### * Silbomyia hoeneana

Taxon classificationAnimaliaDipteraCalliphoridae

Enderlein, 1936

####### Materials.

2♀, T'ai-nan Hsien, Kuan-tzu-ling, 250 m, 06.iv.1965, Rokuro Kano (NSMT); 1♂, Taoyuen, Paling, 09-11.vi.1992, R. Kano (NSMT); 1♀, Kentin, 15.ii.1972, K. Matsuki (NSMT).

#### Tribe Catapicephalini

##### *Catapicephala* Macquart, 1851

###### Catapicephala dasyophthalma

Taxon classificationAnimaliaDipteraCalliphoridae

Villeneuve, 1927

####### Note.

See to James (1977: 532).

###### Catapicephala ruficornis

Taxon classificationAnimaliaDipteraCalliphoridae

Villeneuve, 1927

####### Materials.

1♂, Kaohsiung, Shanping, 640 m, 21–30.iv.1988, C. Young, R. Davidson & J. Rawlins (CMNH); 1♀, Kaohsiung, Shanping, 640 m, 1-10.iv.1988, C. Young, R. Davidson & J. Rawlins (CMNH); 1♀, Chihpen Hot Spring, 3–4.vi.1992, R. Kano (NSMT).

###### Catapicephala splendens

Taxon classificationAnimaliaDipteraCalliphoridae

Macquart, 1851

####### Note.

See to James (1977: 532).

### Subfamily CALLIPHORINAE

#### *Aldrichina* Townsend, 1934

##### Aldrichina grahami

Taxon classificationAnimaliaDipteraCalliphoridae

(Aldrich, 1930)

###### Materials.

1♂ 1♀, Nantou County, Ren'ai Township, Songgang, 2049 m, secondary forest, 16.iv.2013, S. T. Yang (NTU); 2♂ 1♀, Taichung Ctiy, Heping Dist., Huanshan Tribe, Ssuchiehlan Stream, 1832 m, riverbed, 26.i.2013, S. T. Yang (NTU); Mazu, Beigan Is.: 2♂, Lienchiang County, Beigan Township, 100 m, seashore, 12.v.2013, S. T. Yang (NTU); 1♂ 2♀, Alishan, 28–29.v.1992, R. Kano (NSMT); 1♀, nr Huanshan, 2.v.2006, H. Kurahashi (NSMT); 2♂, Alishan-Yushan, 2,600-2,700 m, 31.x.1985, M. Iwasa (NSMT); 1♀, Taoyuen, Paling, 9–11.vi.1992, R. Kano (NSMT); 4♂ 7♀, Oiwake, 4.V.1965, T. Shirozu (NSMT); 6♂ 4♀, Mt. Alishan, 10–13.VII.1964, S. Asahina (NSMT); 2♀, Tattaka, 24.VI.1965, T. Shirozu (NSMT); 1♂, Taichung, Pilushi, 2,200 m, 22–23.v.1988, R. Davidson, C. Young & J. Rawlins (CMNH); 2♂, Alishan-Yushan, 2,600-2,700 m, 31.x.1985, M. Iwasa (NSMT).

#### *Bellardia* Robineau-Desvoidy, 1863

##### Bellardia menechma

Taxon classificationAnimaliaDipteraCalliphoridae

(Séguy, 1934)

###### Materials.

1♂, Hualien Hsien, Juisui, 11.xi.1985, K. Kanmiya (NSMT); 1♂, Chihpen, 10.vi.1972, R. Kano (NSMT); 7♂ 6♀, Juisui, 10–11.xi.1985, K. Kanmiya (NSMT); 2♀, Chihpen, 10.vi.1973, H. M. Lin (IDD); 2♀, Fenchifu, 1,405 m, 2.xi.1985, M. Iwasa (IDD); 1♀, Taichung Hsien, Chichiawanchi, Huanshan, 6.xi.1985, K. Kanmiya (IDD).

##### Bellardia pubescens

Taxon classificationAnimaliaDipteraCalliphoridae

(Macquart, 1851)

###### Materials.

1♂, Chia-i Hsien, Fenchi-Hu, 8.vii.1985, H. Shima (NSMT); 1♀, Nantou Hsien, Janai (Mushia), 28.ix.1965, K. Kaneko (IDD); 1♀, Lishan, 16.ix.1970, S. C. Lien (IDD); 1♀, Chihpen, 10.vi.1972, R. Kano (IDD).

#### *Calliphora* Robineau-Desvoidy, 1830

##### Calliphora nigribarbis

Taxon classificationAnimaliaDipteraCalliphoridae

Vollenhoven, 1863

###### Note.

See to James (1977: 531).

##### Calliphora pattoni

Taxon classificationAnimaliaDipteraCalliphoridae

Aubertin, 1931

###### Materials.

1♂, Taichung City, Heping Dist., Wuling Farm, Taiwan Salmon Observatory Deck, 1800 m, river bed, 23.xii.2012, T. R. Tsai (NTU); 1♀, Taichung Ctiy, Heping Dist., Huanshan Tribe, Ssuchiehlan Stream, 1832 m, riverbed, 26.i.2013, S. T. Yang (NTU); 3♀, Taichung Ctiy, Heping Dist., Siaosyue Mt., Tianchi, 2626 m, coniferous forest, 12.ii.2013, S. T. Yang (NTU); 1♀, Alishan-Yushan, 2,600–2,700 m, 31.x.1985, M. Iwasa (NSMT); 1♂ 2♀, Alishan Mts., 2,270m, 9.IV.1965, R. Kano, Yoshimoto & Parkins (NSMT); 1♂, Tzuchung, 2,370m, 10.IV.1965, S. Ueno (NSMT); 13♂ 5♀, Alishan, 28-29.v.1992, R. Kano & H. M. Lin (NSMT); 1♂, Taichung, Anmashan, 2,230 m, 30.iv–4.v.1990, A. Smetana (CMNH); 1♀, Ren-Ai- He-Huan-Shan, LuoYing Inn, 2,830 m, 22.iii.2011, C. Young (CMNH); 1♀, Alishan-Yushan, 2,600–2,700 m, 31.x.1985, M. Iwasa (NSMT).

##### Calliphora vomitoria

Taxon classificationAnimaliaDipteraCalliphoridae

(Linnaeus, 1758)

###### Materials.

14♂ 10♀, Nantou County, Ren'ai Township, Songgang, 2049 m, secondary forest, 16.iv.2013, S. T. Yang (NTU); 6♀, Taichung Ctiy, Heping Dist., Wuling Farm, Taiwan Salmon Observatory Deck, 1800 m, river bed, 23.xii.2012, T. R. Tsai (NTU); 1♂ 3♀, Taichung City, Heping Dist., Huanshan Tribe, Ssuchiehlan Stream, 1832 m, riverbed, 26.i.2013, S. T. Yang (NTU); 3♀, Taichung Ctiy, Heping Dist., Siaosyue Mt., Tianchi, 2626 m, coniferous forest, 12.ii.2013, S. T. Yang (NTU); 2♂ 1♀, Chiayi County, Alishan Township, Leye Village, Dinghu, 1657 m, secondary forest, 23.ii.2013, S. T. Yang (NTU); 1♂ 1♀, Alishan-Yushan, 2,600-2,700 m, 31.x.1985, M. Iwasa (NSMT); 1♂ 4♀, Taichung, Anmashan, 2,230 m, 30.iv–4.v.1990, A. Smetana (CMNH); 1♂, Anmashan, 2,230 m, 30.iv.–4.v.1990, A. Smetana (CMNH); 1♀, nr Alishan, Hishan, 2,300 m, 30.iv.2006, H. Kurahashi (NSMT); 4♀, Kuanshan trail at Kuanshanchi River, 2,400 m, 20–23.vii.1993, A. Smetana (CMNH); 6♂ 10♀, Alishan, 28–29.v.1992, R. Kano & H. M. Lin (NSMT); 1♂ 1♀, Alishan-Yushan, 2,600–2,700 m, 31.x.1985, M. Iwasa (NSMT); 3♀, Ren-Ai- He-Huan-Shan, LuoYing Inn, 2,830 m, 22.iii.2011, C. Young (CMNH).

#### *Polleniopsis* Townsend, 1917

##### * Polleniopsis dalatensis

Taxon classificationAnimaliaDipteraCalliphoridae

Kurahashi, 1972

###### Materials.

1♂, Nantou County, Ren'ai Township, Jianqing Farm, 26.vii.1961, J. K. Nii (NSMT); 1♂1♀, Fanshan, 1650 m, 6.xi.1985, M. Iwasa (NSMT).

##### Polleniopsis toxopei

Taxon classificationAnimaliaDipteraCalliphoridae

(Senior-White, 1926)

###### Note.

See to James (1977: 540).

#### *Tainanina* Villeneuve, 1926

##### Tainanina pilisquama

Taxon classificationAnimaliaDipteraCalliphoridae

(Senior-White, 1925)

###### Materials.

1♂, Juisui, 10.xi.1985, K. Kanmiya (NSMT); 2♂, Tienshiang, 8.x.1965, K. Kanmiya (NSMT); 1♂, Juisui, 10.xi.1985, K. Kanmiya (NSMT).

##### * Tainanina sarcophagoides

Taxon classificationAnimaliaDipteraCalliphoridae

(Malloch, 1931)

###### Materials.

2♀, Tienshiang, 8.x.1965, K. Kanmiya (NSMT)

### Subfamily MELANOMYINAE

#### *Gymnadichosia* Villeneuve, 1927

##### Gymnadichosia pusilla

Taxon classificationAnimaliaDipteraCalliphoridae

Villeneuve, 1927

###### Materials.

1♂ 1♀, Mt. Alishan, Chun-shan, 2,400 m, 9–10.vii.1985, S. Shinonaga and H. Shima (NSMT);1♂, Chichiawanchi, Huanshan, 6.xi.1985, K. Kanmiya (NSMT).

#### *Paradichosia* Senior-White, 1923

##### Paradichosia crinitarsis

Taxon classificationAnimaliaDipteraCalliphoridae

Villeneuve, 1927

###### Materials.

1♂, Nantou County, Ren'ai Township, Gaofeng, Guantou Mt. (Northeast), 20.v.2013, K. Harusawa (PCKHa); 1♂ 1♀, Pingtung County, Shizi Township, Shuangliu, 189–300 m, secondary forest, 11.v.2013, S. T. Yang (NTU); 1♀, Tsuifeng-Shunkan, 24.vii.1985, H. Shima (NSMT); 1♀, Shanpaling, 26.x.1985, K. Kanmiya (NSMT); 1♂, Hsinchu, Wufeng, 24.xii.1993, C. L. Chung (NSMT).

##### Paradichosia lui sp. n.

Taxon classificationAnimaliaDipteraCalliphoridae

Paradichosia nigricans Villeneuve, 1927

###### Materials.

1♀, Nantou County, Lugu Township, Xitou, 1156 m, secondary forest, 08.iii.2013, S. T. Yang (NTU); 1♂, Taoyuen, Paling, 9-11.vi.1992, R. Kano (NSMT); 1♀, Alishan, 28-29.v.1992, R. Kano (NSMT); 1♀, Fanlu, Kungtien village, 15.iv.1958, S. Y. Liu (NSMT); 1♂, Shihtsao, 24.v.1972, R. Kano (NSMT); 2♂ 1♀, Hohuan-shan, Tsuifeng, 2,400 m, 23-24.vii.1985, S. Shinonaga (NSMT); 1♂, Hsitou, 1,000 m, 1.v.2006, H. Kurahashi (NSMT); 1♀, Alishan, 28-29.v.1992, R. Kano (NSMT).

#### *Pollenomyia* Séguy, 1935

##### Pollenomyia sinensis

Taxon classificationAnimaliaDipteraCalliphoridae

(Séguy)

###### Materials.

1♂, Miaoli, Nanchung, 22.xii.1993, C. L. Chung (NSMT).

#### *Tricycleopsis* Villeneuve, 1927

##### Tricycleopsis paradoxa

Taxon classificationAnimaliaDipteraCalliphoridae

Villeneuve, 1927

###### Note.

See to James (1977: 540).

### Subfamily BENGALIINAE

#### *Bengalia* Robineau-Desvoidy, 1830

##### * Bengalia calilungae

Taxon classificationAnimaliaDipteraCalliphoridae

Rueda, 1985

###### Materials.

1♂, Penpuchi (Honbukei), 21.viii.1980, K. Hara (NSMT).

##### * Bengalia chekiangensis

Taxon classificationAnimaliaDipteraCalliphoridae

Fan, 1965

###### Materials.

3♂ 2♀, Nantou, Wushe, 24.v.1988, C. Young, J. Rawlins & R. Davidson (CMNH); 1♀, Kaohsiung, Shanping, 640 m, 11–20.v.1988, J. Rawlins, C. Young, & R. Davidson (CMNH); 7♂ 9♀, Nantou, Wushe, 24.v.1988, C. Young, J. Rawlins & R. Davidson (CMNH).

##### Bengalia emarginata

Taxon classificationAnimaliaDipteraCalliphoridae

Malloch, 1927

###### Materials.

1♂, Nantou County, Ren'ai Township, Aowanda, 18.v.2013, K. Harusawa (PCKHa); 1♂, Pingtung County, Shizi Township, Shuangliu, 189-300 m, secondary forest, 11.v.2013, S. T. Yang (NTU); 3♂, Taiton, Tsupun, 21.iii.1981, M. Iwasa (NSMT); 1♀, Hotso (Roshan), 30.ix.1965, K. Kaneko (NSMT); 1♀, Kaohsiung, Shanping, 640 m, 11–20.iv.1988, C. Young, J. Rawlins & R. Davidson (CMNH); 1♂, Kotzulin, 850m, 4.IV.1965, S. Ueno (NSMT); 1♂, Alishan, 10–13.VII.1964, S. Asahina (NSMT); 1♂, Fenchihu, 1,370m, 11.IV.1965, B. D. Parkins (NSMT); 1♂, Wulai, 130m, 17.IV.1965, T. Saigusa (NSMT); 2♂, Kenting-kungyuan, 2-3.IV.1965, R. Kano (NSMT); 1♀, Kuantzuling, 250m, 6.IV.1965, R. Kano (NSMT); 3♂ 1♀, Nanzan-kei, 30.IV.1965, 8.V.1965, T. Shirozu (NSMT); 1♂, Chihpen Hot Spring, 3–4.vi.1992, R. Kano (NSMT); 1♂, Chung-Hsien University, Huei-sun Forest, 21.v.1992, R. Kano (NSMT); 1♂, Kenting Park, 1–2.vi.1992, R. Kano (NSMT); 1♀, Nantou, Wushe, 24.v.1988, C. Young, J. Rawlins & R. Davidson (CMNH).

##### Bengalia escheri

Taxon classificationAnimaliaDipteraCalliphoridae

Bezzi, 1913

###### Materials.

1♀, Nantou County, Ren'ai Township, Aowanda, 18.v.2013, K. Harusawa (PCKHa); 3♂, Taipei City, Neihu Dist., Daluntou Mt., 352 m, secondary forest, 19.v.2013, S. T. Yang (NTU); 1♂, New Taipei City, Wulai Dist., Hsiaoi Village, Tonghou, 233 m, dump (at light), 30.v.2012, S. T. Yang (NTU); 1♀, Kaohsiung, Shanping, 640 m, 21–30.iv.1988, C. Young, R. Davidson & J. Rawlins (CMNH); 1♂, Fenchihu, 1,370m, 10.IV.1965, R. Kano (NSMT); 1♂ 1♀, Fenchihu, 4.IV.1965, 12.IV.1965, R. Kano (NSMT); 1♀, Ten-chih, 23-04-03 N, 120-45-13 E, 1,550 m, 23.viii.1996, C. W. Young (CMNH); 1♂, Taoyuen, Paling, 9–11.vi.1992, R. Kano (NSMT); 2♀, Lushan, 1,000m, 24-25.VII.1985, S. Shinonaga (NSMT); 1♂ 1♀, Kukan, 3.XI.1985, K. Kanmiya (NSMT); 1♂, Nanshanchi, 31.iii.1996, R. Matsumoto (BLKU); 1♀, Wulai-hsiang, Fushan, 400–600 m, 28.xi.1997, T. Tachi (BLKU); 8♂ 1♀, Fushan B. G., 600 m, 3–6.v.2006, H. Kurahashi (NSMT); 1♂, Chiapaotai Park, 4.x.1985, K. Kanmiya (NSMT).

##### Bengalia fuscipennis

Taxon classificationAnimaliaDipteraCalliphoridae

Bezzi, 1913

###### Materials.

1♂, Nantou County, Puli Township, Shizitou, at light, 20.v.2013, K. Harusawa (PCKHa); 1♀, Nantou County, Ren'ai Township, Gaofeng, Guantou Mt. (Northeast), 20.v.2013, K. Harusawa (PCKHa); 2♂, New Taipei City, Wulai Dist., Hsiaoi Village, Tonghou, 233 m, dump (at light), 30.v.2012, S. T. Yang (NTU); 16♂ 7♀, Fushan B. G., 600 m, 3-6.v.2006, H. Kurahashi (NSMT); 2♀, Wulai, 150m,17.IV.1965, 20.X.1985, R. Kano & K. Kanmiya (NSMT); 1♀, Lafu, 350m, 27.X.1985, R. Kano (NSMT); 1♂ 3♀, Fenchifu, 1,400m, 5.VII.1985, 8.VII.1985, S. Shinonaga & H. Shima (NSMT); 5♂ 2♀, Lenai, 1,000m, 23.VII.1985, S. Shinonaga (NSMT); 1♀, Tattaka, 29.VI.1965, T. Shirozu (NSMT); 2♀, Kaohsiung, Shanping, 640 m, 21–30.iv.1988, C. Young, J. Rawlins & R. Davidson (CMNH); 2♂, Chiapaotai Park, 4.x.1985, K. Kanmiya (NSMT); 1♂ 5♀, Kaohsiung, Shanping, 640 m, 21-30.iv.1988, C. Young, R. Davidson & J. Rawlins (CMNH); 2♀, Kaohsiung, Shanping, 640 m, 11–20.iv.1988, C. Young, R. Davidson & J. Rawlins (CMNH); 2♂ 1♀, Chung-Hsien University, Huei-sun Forest, 21.v.1992, R. Kano (NSMT).

##### Bengalia taksina

Taxon classificationAnimaliaDipteraCalliphoridae

(Lehrer, 2005)

###### Materials.

1♀, Nanshanchi, 31.iii.1996, R. Matsumoto (BLKU); 5♂ 5♀, Nantou, Wushe, 24.v.1988, C. Young, J. Rawlins & R. Davidson (CMNH); 1♂, Kaohsiung, Shanping, 640 m, 21–30.iv.1988, C. Young, R. Davidson & J. Rawlins (CMNH); 1♂, Nantou, Wushe, 640 m, 24.v.1988, C. Young, J. Rawlins & R. Davidson (CMNH); 2♀, Taoyuen, Paling, 9–11.vi.1992, R. Kano (NSMT); 3♂ 1♀, Kuantzuling, 28.v.1992, R. Kano (NSMT); 2♂ 1♀, Puli, Nanshanhsi, 23–24.v.1992, R. Kano (NSMT); 2♂ 1♀, Chung-Hsien University, Huei-sun Forest, 21.v.1992, R. Kano (NSMT).

##### Bengalia torosa

Taxon classificationAnimaliaDipteraCalliphoridae

(Wiedemann, 1819)

###### Materials.

2♀, Kaohsiung, Shanping, 640 m, 1–10.iv.1988, R. Davidson, J. Rawlins & C. Young (CMNH); 1♂, Kaohsiung, Shanping, 640 m, 21-30.iv.1988, C. Young, J. Rawlins & R. Davidson (CMNH).

##### Bengalia varicolor

Taxon classificationAnimaliaDipteraCalliphoridae

(Fabricius, 1805)

###### Materials.

1♂ 1♀, Nantou County, Ren'ai Township, Aowanda, 18.v.2013, K. Harusawa (PCKHa); 2♀, Nantou County, Ren'ai Township, Aowanda, 21.v.2013, K. Harusawa (PCKHa); 13♂ 24♀, Jenai, Chinglin, 27.V.1972, R. Kano (NSMT); 1♂, Nanzan-kei, 26.VI.1965, T. Shirozu (NSMT); 1♂, Kenting-kung-yuan, 2.IV.1965, R. Kano (NSMT); 1♀, Tienshiang, 8.X.1985, K. Kanmiya (NSMT); 2♂ 1♀, Hotso (Roshan), 30.IX.1965, K. Kaneko (NSMT).

### Subfamily LUCILIINAE

#### *Hemipyrellia* Townsend, 1918

##### Hemipyrellia ligurriens

Taxon classificationAnimaliaDipteraCalliphoridae

(Wiedemann, 1830)

###### Materials.

1♂, Nantou County, Puli Township, Qianxi, 20.v.2013, K. Harusawa (PCKHa); 3♂ 7♀, Yilan County, Nan'ao Township, 22 m, secondary forest; eggs collected, 7.iii.2013 eclosion, 16.ii.2013, S. T. Yang (NTU); 4♀, Tainan City, Baihe Dist., Guanziling, Lingding Park, 322 m, secondary forest, 21.iii.2013, S. T. Yang (NTU); 1♀, New Taipei City, Shimen Dist., Linshan Cape, 20 m, seashore, 29.xii.2012, S. T. Yang (NTU); 1♀, New Taipei City, Xindian Dist., Zhitan Dam, 57 m, riverside, 27.xii.2012, S. T. Yang (NTU); 1♀, Hualien County, Xiulin Township, Fushi, Changuang Temple, 120 m, monsoon rainforest, 09.v.2013, S. T. Yang (NTU); Orchid Is.: 1♂, Taitung County, Lanyu Township, Hongtou, Weather Station, 231 m, tropical rainforest, 01.vi.2013, S. T. Yang (NTU); Orchid Is.: 1♀, Taitung County, Lanyu Township, Zhong'ai Bridge, 53 m, tropical rainforest, 01.vi.2013, S. T. Yang (NTU); 18♂ 22♀, Hualien County, Shoufeng Township, Pinghe, 41 m, plain; eggs collected, 7.iii.2013 eclosion, 16.ii.2013, S. T. Yang (NTU); Mazu, Beigan Is.: 3♂ 3♀, Lienchiang County, Beigan Township, 100 m, seashore, 12.v.2013, S. T. Yang (NTU); 2♂ 1♀, Kaohsiung City, Liugui Dist., Tsaidie Valley, 366 m, secondary forest, 31.iii.2013, S. T. Yang (NTU); 5♂ 12♀, New Taipei City, Wulai Dist., Zhongzhi, 109 m, riverside; eggs collected, 18.i.2013 eclosion, 27.xii.2012, S. T. Yang (NTU); 10♂ 5♀, Hualien County, Xincheng Township, Qixingtan, Sihba Height, 41 m, grassland, 09.v.2013, S. T. Yang (NTU); 5♀, Hualien County, Xincheng Township, Qixingtan, 14 m, seashore, 09.v.2013, S. T. Yang (NTU); 5♂ 4♀, Pingtung County, Sangdimen Township, Sandi Village, 381 m, secondary forest, 2.iv.2013, S. T. Yang (NTU); 1♀, New Taipei City, Zhonghe Dist., Yuantong Temple, 173 m, secondary forest, 25.v.2013, S. T. Yang (NTU); 2♂ 2♀, Yilan County, Toucheng Township, Beiguan, 34 m, seashore, 04.iv.2013, S. T. Yang (NTU); 2♂ 1♀, Chiayi County, Alishan Township, Shanmei, Tanayiku, 450 m, secondary forest, 24.ii.2013, S. T. Yang (NTU); 11♂, Taitung County, Haiduan Township, Xinwulu, 390 m, secondary forest, 30.v.2013, S. T. Yang (NTU); 1♂ 3♀, Taitung County, Luye Township, Ruiyuan, 198 m, farmland, 30.v.2013, S. T. Yang (NTU); 2♂, Taitung County, Luye Township, Luye High Terrace, 353 m, grassland, 30.v.2013, S. T. Yang (NTU); 2♂ 2♀, Taitung County, Dawu Township, Dawu, 85 m, secondary forest, 18.iv.2013, S. T. Yang (NTU); Liuqiu Is.: 2♂ 1♀, Pingtung County, Liuqiu Township, Geban Bay, 10 m, seashore, 04.v.2013, S. T. Yang (NTU); Liuqiu Is.: 8♂ 2♀, Pingtung County, Liuqiu Township, Shanfu, 25 m, seashore, 04.v.2013, S. T. Yang (NTU); Liuqiu Is.: 3♂ 6♀, Pingtung County, Manzhou Township, Qikong Waterfall, 40–125 m, secondary forest, 24.i.2013, S. T. Yang (NTU); 5♂ 2♀, Kaohsiung City, Jiaxian Dist., Jiaxian, 278 m, secondary forest, 12.v.2013, S. T. Yang (NTU); 4♂ 13♀, Taipei City, Da'an Dist., NTU Agricultural Entomology Building, 12 m, orchard; eggs collected, 05.ii.2013 eclosion, 22.i.2013, S. T. Yang (NTU); 1♀, Taipei City, Da'an Dist., NTU Agricultural Entomology Building, 12 m, orchard, 22.i.2013, S. T. Yang (NTU); 1♂, Taipei City, Da'an Dist., NTU Agricultural Entomology Building, 12 m, orchard, 01.iv.2013, S. T. Yang (NTU); 19♂ 11♀, Taipei City, Da'an Dist., NTU Agricultural Entomology Building, 12 m, orchard; eggs collected, 20.xii.2012 eclosion, 19.viii.2009, S. T. Yang (NTU); 7♂ 1♀, Taipei City, Da'an Dist., Fuzhoushan Park, 71 m, secondary forest, 17.iii.2013, S. T. Yang (NTU); 2♀, Taipei City, Da'an Dist., Fuzhoushan Park, 100 m, secondary forest, 17.iii.2013, S. T. Yang (NTU); LANHSU IS.: 1♂ 1♀, 17–19.VII.12985, S. Shinonaga (NSMT); 1♀, Wulai, 4.VII.1985, S. Shinonaga (NSMT); 1♂, Kenting Park, 1–2.vi.1992, R. Kano (NSMT); 1♂ 1♀, Kuatsuling, ll.VII.1985, S. Shinonaga (NSMT); 1♂, Shu Lin, 16.VIII.1987, I. Togashi (IAC); 1♂ 1♀, Santimen, 13.VII.1985, S. Shinonaga (NSMT); 1♂, Juisui, 10.XI.1985, K. Kanmiya (NSMT); 1♂ 1♀, Taipei City, 20–17.III.1965, R. Kano (NSMT); 3♂, Liuknei, 12.VII.1985, H. Shima (NSMT); 1♂ 1♀, Kuatsuling, 10–11.VII.1985, H. Shima (NSMT); 8♂ 1♀, Chihpen, 15.XI.1985, K. Kanmiya (NSMT); 5♂ 3♀, Antung, 12.XI.1985, K. Kanmiya (NSMT); 1♂ 2♀, Chihpen Hot Spring, 3–4.vi.1992, H. M. Lin (NSMT); 1♂, Jiji (Chichi), 30.iv.2006, H. Kurahashi (NSMT); 3♂, Kuantzuling, 26.v.1992, R. Kano (NSMT).

#### *Lucilia* Robineau-Desvoidy, 1830

##### Lucilia bazini

Taxon classificationAnimaliaDipteraCalliphoridae

Séguy, 1934

###### Materials.

Mazu, Beigan Is.: 1♀, Lienchiang County, Beigan Township, 100 m, seashore, 12.v.2013, Y. C. Yu (NTU); 1♀, Kaohsiung, Shanping, 640 m, 23–31.iii.1988, R. Davidson, J. Rawlins & C. Young (CMNH); 1♂ 1♀, Kaohsiung, Shanping, 640 m, 21–30.iv.1988, R. Davidson, J. Rawlins & C. Young (CMNH).

##### Lucilia calviceps

Taxon classificationAnimaliaDipteraCalliphoridae

Bezzi, 1927

###### Materials.

2♂, Chihpen, 20.VII.1985, 15.XI.1985, S. Shinonaga (NSMT); 1♂, Chuchi, 120m, 14.IV.1965, R. Kano (NSMT); 1♀, Yuankan-Tsuifen, 23.VII.1985, H. Shima (NSMT).

##### Lucilia cuprina

Taxon classificationAnimaliaDipteraCalliphoridae

(Wiedemann, 1830)

###### Materials.

1♂, Hualien County, Xincheng Township, Qixingtan, 14 m, seashore, 09.v.2013, S. T. Yang (NTU); 1♀, Hualien County, Xincheng Township, Qixingtan, Sihba Height, 41 m, grassland, 09.v.2013, S. T. Yang (NTU); 1♀, Chiayi County, Alishan Township, Shanmei, Tanayiku, 450 m, secondary forest, 24.ii.2013, S. T. Yang (NTU); 1♀, Fenchifu, 1,400m, 8.VII.1985, S. Shinonaga (NSMT); 1♂, Kwantyling, 11.VII.1985, S. Shinonaga (NSMT); 1♀, Shanpaling, 27.x.1985, K. Kanmiya (NSMT); 1♀, Taipei, 30.III.1965, R. Kano (NSMT); 1♀, Taipei, 4.III.1981, M. Iwasa (NSMT); 1♀, Laanung, 11.VII.1985, S. Shinonaga (NSMT); 1♂ 1♀, San Palin, 1,100m, 25.X.1985, R. Kano (NSMT); 1♂, Pinton, 6.III.1981, M. Iwasa (NSMT); 2♂ 2♀, Shanpaling, 27.X.1985, K. Kanmiya (NSMT); 1♂, Kwantyling, 11.VII.1985, S. Shinonaga (NSMT); 1♀, Shanpaling, 27.x.1985, K. Kanmiya (NSMT).

##### Lucilia hainanensis

Taxon classificationAnimaliaDipteraCalliphoridae

Fan, 1965

###### Materials.

2♂, Nantou County, Ren'ai Township, Gaofeng, Guantou Mt. (Northeast), 20.v.2013, K. Harusawa (PCKHa); 2♂ 3♀, Pingtung County, Shizi Township, Shuangliu, 189-300 m, secondary forest, 11.v.2013, S. T. Yang (NTU); 4♀, Taitung County, Haiduan Township, Xinwulu, 390 m, secondary forest, 30.v.2013, S. T. Yang (NTU); 5♀, Taitung County, Dawu Township, Dawu, 85 m, secondary forest, 18.iv.2013, S. T. Yang (NTU); 2♀, Taitung County, Haiduan Township, Xiama, 790 m, secondary forest, 30.v.2013, S. T. Yang (NTU); 1♀, Chiayi County, Alishan Township, Shanmei, Tanayiku, 450 m, secondary forest, 24.ii.2013, S. T. Yang (NTU); 2♂ 30♀, Kaohsiung City, Liugui Dist., Tsaidie Valley, 366 m, secondary forest, 31.iii.2013, S. T. Yang (NTU); 2♂ 17♀, Pingtung County, Sangdimen Township, Sandi Village, 381 m, secondary forest, 2.iv.2013, S. T. Yang (NTU); 20♂ 10♀, Kaohsiung, Shanping, 640 m, 11-20.iv.1988, 21–30.iv.1988, 1–10.v.1988, R. Davidson, J. Rawlins & C. Young (CMNH); 1♀, Taichung, Anmashan, 2,230 m, 30.iv–4.v.1990, A. Smetana (CMNH); 1♂, Anmashan, 2,230 m, 30.iv.–4.v.1990, A. Smetaana (CMNH).

##### Lucilia papuensis

Taxon classificationAnimaliaDipteraCalliphoridae

Macquart, 1842

###### Materials.

1♀, Nantou County, Lugu Township, Xitou, 1156 m, secondary forest, 08.iii.2013, S. T. Yang (NTU); 1♀, Taitung County, Beinan Township, Jhihben, 209 m, secondary forest, 15.ii.2013, S. T. Yang (NTU); 1♀, Nantou County, Yuchi Township, Sun Moon Lake, 803 m, lakeside, 20.i.2013, S. T. Yang (NTU); 1♀, Taitung County, Beinan Township, Jhihben, 209 m, secondary forest, 15.ii.2013, S. T. Yang (NTU); 1♀, Juisui, 11.XI.1985, K. Kanmiya (NSMT); 3♀, Lushan, 1,000m, 24–25.VII.1985, S. Shinonaga (NSMT); 2♀, Chihpen, 15.XI.1985, K. Kanmiya (NSMT); 2♀, Ten-chih, 23-04-03 N, 120-45-13 E, 1,550 m, 23.viii.1996, C. W. Young (CMNH); 1♀, Kuantzuling, 250m, 6.IV.1965, R. Kano (NSMT); 1♀, Lenai, 1,000m, 23.VII.1985, S. Shinonaga (NSMT); 1♀, Fushan B. G., 600 m, 3–6.v.2006, H. Kurahashi (NSMT); 1♀, Nanshanchi, 1.v.2006, H. Kurahashi (NSMT); 6♀, Kaohsiung, Shanping, 640 m, 1–10.v.1988, R. Davidson, J. Rawlins & C. Young (CMNH); 1♀, Kaohsiung, Shanping, 640 m, 11–20.iv.1988, J. Rawlins, C. Young, R. Davidson (CMNH); 3♀, Meifeng, 2,130 m, 10–17.vii.1993, A. Smetana (CMNH); 6♀, Taoyuen, Paling, 9–11.vi.1992, R. Kano (NSMT); 1♀, Taichung, Anmashan, 2,230 m, 30.iv.1990, A. Smetana (CMNH).

##### Lucilia porphyrina

Taxon classificationAnimaliaDipteraCalliphoridae

(Walker, 1857)

###### Materials.

1♂, Nantou County, Ren'ai Township, Aowanda, 21.v.2013, K. Harusawa (PCKHa); 4♀, Nantou County, Ren'ai Township, Aowanda, 18.v.2013, K. Harusawa (PCKHa); 2♀, Nantou County, Ren'ai Township, Gaofeng, Guantou Mt. (Northeast), 20.v.2013, K. Harusawa (PCKHa); 21♂ 23♀, Yilan County, Datong Township, Cilan, 394 m, secondary forest; eggs collected, 11.ii.2013 eclosion, 27.i.2013, S. T. Yang (NTU); 1♂ 3♀, Chiayi County, Alishan Township, Shanmei, Tanayiku, 450 m, secondary forest, 24.ii.2013, S. T. Yang (NTU); 3♀, Taipei City, Neihu Dist., Daluntou Mt., 352 m, secondary forest, 19.v.2013, S. T. Yang (NTU); 9♂ 46♀, Nantou County, Lugu Township, Xitou, 1156 m, secondary forest, 08.iii.2013, S. T. Yang (NTU); 19♂ 28♀, Nantou County, Lugu Township, Xitou, 1156 m, secondary forest; eggs collected, 26.iii.2013 eclosion, 08.iii.2013, S. T. Yang (NTU); 2♂ 2♀, Taipei City, Beitou Dist., Yangmingshan Anbu, 837 m, arrow bamboo forest, 31.i.2013, S. T. Yang (NTU); 8♂ 19♀, New Taipei City, Wulai Dist., Xinxian Village, 219 m, riverside, 16.iii.2013, S. T. Yang (NTU); 7♀, New Taipei City, Wulai Dist., Hsiaoi Village, Tonghou, 233 m, riverside, 27.xii.2012, S. T. Yang (NTU); 6♂ 7♀, New Taipei City, Wulai Dist., Hsiaoi Village, Tonghou, 233 m, riverside; eggs collected, 18.i.2013 eclosion, 27.xii.2012, S. T. Yang (NTU); 9♀, Taoyuan County, Fuxing Township, Baling, 640 m, secondary forest, 07.i.2013, S. T. Yang (NTU); 1♀, Nantou County, Ren'ai Township, Meifeng Farm, 2100 m, secondary forest, 24.x.2009, S. T. Yang (NTU); 1♂, Tainan City, Baihe Dist., Guanziling, Red-Leaf Park, 357 m, secondary forest, 21.iii.2013, S. T. Yang (NTU); 12♂ 9♀, New Taipei City, Wugu Dist., Guanyin Mt., Yinghan Peak, 359-611 m, secondary forest, 30.iv.2013, S. T. Yang (NTU); 2♀, Taichung City, Heping Dist., Guguan, Songhe Tribe, 700 m, secondary forest, 14.iv.2013, S. T. Yang (NTU); 1♂, Taichung City, Heping Dist., Huanshan Tribe, Ssuchiehlan Stream, 1832 m, riverbed; larvae collected, 09.ii.2013 eclosion, 26.i.2013, S. T. Yang (NTU); 1♂, Nantou County, Ren'ai Township, Qingjing Farm, 1911 m, secondary forest, 16.iv.2013, S. T. Yang (NTU); 2♀, New Taipei City, Xindian Dist., Zhitan Dam, 57 m, riverside, 27.xii.2012, S. T. Yang (NTU); 7♀, Pingtung County, Shizi Township, Shuangliu, 352 m, secondary forest, 11.v.2013, S. T. Yang (NTU); 34♂ 35♀, Yilan County, Su'ao Township, 28 m, rural area; eggs collected, 7.iii.2013 eclosion, 16.ii.2013, S. T. Yang (NTU); 11♂ 12♀, Yilan County, Nan'ao Township, 22 m, secondary forest; eggs collected, 7.iii.2013 eclosion, 16.ii.2013, S. T. Yang (NTU); 2♀, Yilan County, Nan'ao Township, 22 m, secondary forest, 16.ii.2013, S. T. Yang (NTU); 4♀, New Taipei City, Wulai Dist., Zhongzhi, 109 m, riverside,27.xii.2012, S. T. Yang (NTU); 5♂ 24♀, New Taipei City, Wulai Dist., Zhongzhi, 109 m, riverside; eggs collected, 18.i.2013 eclosion,27.xii.2012, S. T. Yang (NTU); 10♀, Chiayi County, Alishan Township, Leye Village, Dinghu, 1657 m, secondary forest, 23.ii.2013, S. T. Yang (NTU); 21♂ 25♀, Chiayi County, Alishan Township, Leye Village, Dinghu, 1657 m, secondary forest; eggs collected, 18.iii.2013 eclosion, 23.ii.2013, S. T. Yang (NTU); 14♂ 19♀, Taipei City, Wenshan Dist., Saint's Alp, 144 m, secondary forest; eggs collected, 08.i.2013 eclosion, 21.xii.2013, S. T. Yang (NTU); 2♂ 19♀, Taipei City, Wenshan Dist., Saint's Alp, 144 m, secondary forest, 21.xii.2013, S. T. Yang (NTU); 1♀, Taipei City, Wenshan Dist., Saint's Alp, 144 m, secondary forest, 20.xii.2013, S. T. Yang (NTU); 2♀, New Taipei City, Ruifang Dist., Jinguashih, 360 m, house, 05.ii.2013, T. H. Wu (NTU); 9♂ 22♀, Taipei City, Da'an Dist., NTU Agricultural Entomology Building, 12 m, orchard; eggs collected, 05.ii.2013 eclosion,22.i.2013, S. T. Yang (NTU); 1♂ 20♀, Yilan County, Toucheng Township, Beiguan, 34 m, seashore, 04.iv.2013, S. T. Yang (NTU); 1♂ 9♀, New Taipei City, Shimen Dist., Linshan Cape, 20 m, seashore, 29.xii.2012, S. T. Yang (NTU); 13♂ 17♀, New Taipei City, Shimen Dist., Linshan Cape, 20 m, seashore; eggs collected, 21.i.2013 eclosion, 29.xii.2012, S. T. Yang (NTU); 1♂ 17♀, Yilan County, Toucheng Township, Yingzi Mt., 936 m, bush, 27.v.2013, S. T. Yang (NTU); 1♀, Taitung County, Haiduan Township, Xiama, 790 m, secondary forest, 30.v.2013, S. T. Yang (NTU); Mazu, Beigan Is.: 1♂ 1♀, Lienchiang County, Beigan Township, 100 m, seashore, 12.v.2013, Y. C. Yu (NTU); 9♂ 10♀, New Taipei City, Pingxi Dist., Lingjiao, 213 m, secondary forest; eggs collected, 19.iii.2013 eclosion, 28.ii.2013, S. T. Yang (NTU); 2♂ 15♀, Chiayi County, Alishan Township, Leye Village, Zhunghu, 1383 m, secondary forest, 23.ii.2013, S. T. Yang (NTU); 1♂, Taitung County, Beinan Township, Jhihben, 209 m, secondary forest, 15.ii.2013, S. T. Yang (NTU); 1♂, New Taipei City, Shiding Dist., Tanyao, 300 m, lakeshore, 2.ii.2013, S. T. Yang (NTU); 2♂ 3♀, Taipei City, Da'an Dist., Fuzhoushan Park, 71 m, secondary forest, 17.iii.2013, S. T. Yang (NTU); 2♂ 9♀, New Taipei City, Ruifang Dist., Jinguashih, Chahu Mt., 416 m, bush, 01.ii.2013, S. T. Yang (NTU); 21♀, New Taipei City, Ruifang Dist., Jinguashih, Chahu Mt., 343 m, bush, 01.ii.2013, S. T. Yang (NTU); 15♂ 36♀, New Taipei City, Ruifang Dist., Jinguashih, Chahu Mt., 343 m,bush; eggs collected, 19.ii.2013 eclosion,01.ii.2013, S. T. Yang (NTU); 30♂ 28♀, Taipei City, Wenshan Dist., Saint's Alp, 144 m, secondary forest; eggs collected, 08.i.2013 eclosion, 20.xii.2013, S. T. Yang (NTU); 20♂ 17♀, Yilan County, Datong Township, 144 m, secondary forest; eggs collected, 11.ii.2013 eclosion, 27.i.2013, S. T. Yang (NTU); 1♀, Hualien County, Guangfu Township, 143 m,plain,16.ii.2013, S. T. Yang (NTU); 3♂ 8♀, Chiayi County, Zhuqi Township, Shizhao, 774 m, secondary forest; eggs collected, 18.iii.2013 eclosion, 23.ii.2013, S. T. Yang (NTU); 4♀, New Taipei City, Gongliao Dist., Shuangyu, 16 m, secondary forest; eggs collected, 19.iii.2013 eclosion, 28.ii.2013, S. T. Yang (NTU); 2♀, Chiayi County, Alishan Township, Dingshizhao, 1446 m, secondary forest, 24.ii.2013, S. T. Yang (NTU); 6♂ 10♀, New Taipei City, Sanzhi Dist., Beixinzhuang, 327 m, secondary forest, 31.i.2013, S. T. Yang (NTU); 9♀, New Taipei City, Gongliao Dist., Santiago Cape, 97 m, bush, 28.ii.2013, S. T. Yang (NTU); 21♂ 18♀, Yilan County, Su'ao Township, Nanfang'ao, 336 m, seashore; eggs collected, 7.iii.2013 eclosion, 16.ii.2013, S. T. Yang (NTU); 1♂ 2♀, New Taipei City, Xindian Dist., Qingtan, Sishifen, 419 m, secondary forest, 07.ii.2013, S. T. Yang (NTU); 16♂ 22♀, Chiayi County, Fanlu Township, Xiding, 1247 m, secondary forest; eggs collected, 18.iii.2013 eclosion, 23.ii.2013, S. T. Yang (NTU); 6♂ 2♀, Hualien County, Xiulin Township, Chongde, 73 m, seashore, 16.ii.2013, S. T. Yang (NTU); 4♀, Hualien County, Xiulin Township, Fushi, Changuang Temple, 120 m, monsoon rainforest, 09.v.2013, S. T. Yang (NTU); 1♂ 4♀, nr Huanshan, 2.v.2006, H. Kurahashi (NSMT); 8♂ 14♀, Fushan B. G., 600 m, 3-6.v.2006, H. Kurahashi (NSMT); 3♂ 4♀, Nanshanchi, 1.v.2006, H. Kurahashi (NSMT); 1♂, Kaohsiung, Shanping, 640 m, 21-30.iv.1988, C. Young, R. Davidson & J. Rawlins (CMNH); 1♂ 7♀, Kaohsiung, Shanping, 640 m, 21-30.iv.1988, 11-20.v.1988, R. Davidson, J. Rawlins & C. Young (CMNH); 3♂ 5♀, Hsito, 1,000 m, 1.v.2006, H. Kurahashi (NSMT); 2♂ 6♀, Taichung, Anmashan, 2,230 m, 30.iv–4.v.1990, A. Smetana (CMNH); 2♂, Meifend, 2,130 m, 10–17.vii.1993, A. Smetana (CMNH); 1♂ 2♀, Kaoshiung, Shanping, 640 m, 11–20.iv.1988, J. Rawlins, C. Young and R. Davidson (CMNH); 1♀, Kuanshan trail at Kuanshanchi River, 2,400 m, 20–23.vii.1993, A. Smetana (CMNH); 1♂, Anmashan, 2,230 m, 30.iv.–4.v.1990, A. Smetaana (CMNH); 2♂ 2♀, Ten-chih, 23-04-03N, 120-45-13E, 1,550 m, 23.viii.1996, C. W. Young (CMNH); 1♀, Puri, 27.III.1981, M. Iwasa (NSMT); 2♂ 1♀, Hohuan-shan, Tsuifeng, 2,400m, 23-24.VII.1985, S. Shinonaga (NSMT); 1♂, Tsuifeng, 19.vi.1970, H. Kurahashi (NSMT); 3♂ 1♀, Fenchifu, 1,400m,12.IV.1965, 8.VII.1985, 20.XI.1985, K. Kanmiya, R. Kano & H. M. Lin (NSMT); 1♀, Hohuan-shan, Yuankang, 2,700m, 23.VII.1985, S. Shinonaga (NSMT); 2♀, Huanshan, Chichiawanchi, 4.XI.1985, K. Kanmiya, R. Kano & H. M. Lin (NSMT); 7♀, Alishan, 2,400m, 9-10.VII.1985, H. M. Lin & H. Shima (NSMT); 3♀, Mt. Yangming shan, 450m, 28.III.1965, 8.VII.1985, R. Kano & S. Shinonaga (NSMT); 1♀, Chiapaotai, 4.XI.1985, K. Kanmiya, R. Kano & H. M. Lin (NSMT); 3♂ 15♀, Lala shan, 1,400–1,600m, 25-26.X.1985, R. Kano & H. M. Lin (NSMT); 1♀, Puri, Rushan, 26.III.1981, M. Iwasa (NSMT); 1♂ 4♀, Shanpaling, 26.X.1985, K. Kanmiya (NSMT); 1♀, Taiton, Tsupun, 21.III.1981, M. Iwasa (NSMT); 1♀, Paling, Taoyuen, 9–11.vi.1992, R. Kano (NSMT); 1♂ 8♀, Alishan, 28–29.v.1992, R. Kano (NSMT); 2♂ 17♀, Chung-Hsien University, Huei-sun Forest, 21.v.1992, R. Kano (NSMT); 3♂ 4♀, Puli, Nanshanhsi, 23–24.v.1992, H. M. Lin (NSMT); 5♂ 24♀, Taoyuen, Paling, 9–11.vi.1992, R. Kano (NSMT); 1♀, Kenting Park, 1–2.vi.1992, R. Kano (NSMT); 2♂ 6♀, Taoyuen, Lalashan, 12.vi.1992, H. M. Lin (NSMT); 1♀, Tattaka, 1.VI.1965, T. Shirozu (NSMT).

##### Lucilia sinensis

Taxon classificationAnimaliaDipteraCalliphoridae

Aubertin, 1933

###### Materials.

1♀, Nanzan-kei, 27.V.1965, T. Shirozu (NSMT); 1♂, Hohuan-shan, Yuankang, 2,700m, 23.VII.1985, S. Shinonaga (NSMT); 2♀, Kenting Park, 17.X.1985, K. Kanmiya (NSMT); 1♀, Mt. Yuishan, 2,700-3,500m, 6-7.VII.1985, S. Shinonaga (NSMT).

##### Lucilia sericata

Taxon classificationAnimaliaDipteraCalliphoridae

(Meigen, 1826)

###### Materials.

6♂ 4♀, Taichung City, Heping Dist., Huanshan Tribe, Ssuchiehlan Stream, 1832 m, riverbed; larvae collected, 09.ii.2013 eclosion, 26.i.2013, S. T. Yang (NTU); 1♀, Fenchifu, 1,370m, 11.IV.1965, R. Kano (NSMT); 1♂, Huanshan, Chichiawanchi, 6.XI.1985, K. Kanmiya, R. Kano & H. M. Lin (NSMT); 1♂, Taipei City, 30.III.1965, S. Miyamoto (NSMT).

##### Lucilia taiwanica

Taxon classificationAnimaliaDipteraCalliphoridae

Kurahashi & Kano, 1995

###### Materials.

2♂, Nantou County, Ren'ai Township, Aowanda, 18.v.2013, K. Harusawa (PCKHa); 2♂, Nantou County, Ren'ai Township, Songgang, 2049 m, secondary forest, 16.iv.2013, S. T. Yang (NTU); 1♀, Nantou County, Ren'ai Township, Meifeng Farm, 2100 m, secondary forest, 24.x.2009, S. T. Yang (NTU); 1♀, Nantou County, Lugu Township, Xitou, 1156 m, secondary forest, 08.iii.2013, S. T. Yang (NTU); 1♀, New Taipei City, Wugu Dist., Guanyin Mt., Yinghan Peak, 359–611 m, secondary forest, 30.iv.2013, S. T. Yang (NTU); 4♀, Fushan B. G., 600 m, 3–6.v.2006, H. Kurahashi (NSMT); 2♀, Ten-chih, 23-04-03 N, 120-45-13 E, 1,550 m, 23.viii.1996, C. W. Young (CMNH).

### Subfamily PHUMOSIINAE

#### *Caiusa* Surcouf, 1919

##### Caiusa indica

Taxon classificationAnimaliaDipteraCalliphoridae

Surcouf, 1914

###### Materials.

1♂, Shanping, 640 m, 21-30.iv.1988, C. Young, R. Davidson and J. Rawlins (CMNH).

##### Caiusa testacea

Taxon classificationAnimaliaDipteraCalliphoridae

Senior-White, 1923

###### Note.

See to Hennig (1941: 180).

##### Caiusa sp.

Taxon classificationAnimaliaDipteraCalliphoridae

###### Materials.

7♂ 4♀, New Taipei City, Xindian Dist., Sikanshui, 500 m, secondary forest, 22.ii.2013, Y. R. Huang (NTU); 1♀, New Taipei City, Xindian Dist., Sikanshui, 500 m, secondary forest, 28.ii.2013, Y. R. Huang (NTU); 2♂, Taitung County, Haiduan Township, Xiama, 790 m, secondary forest, 30.v.2013, S. T. Yang (NTU).

### Subfamily POLLENIINAE

#### *Dexopollenia* Townsend, 1917

##### Dexopollenia flava

Taxon classificationAnimaliaDipteraCalliphoridae

(Aldrich, 1930)

###### Materials.

1♂, Taichung, Pilushi, 2,200 m, 22-23.v.1988, R. Davidson, C. Young & J. Rawlins (CMNH); 1♂, Fanshan, 1,650 m, 5.xi.1985, M. Iwasa (IDD).

##### Dexopollenia luteola

Taxon classificationAnimaliaDipteraCalliphoridae

(Villeneuve, 1927)

###### Note.

See to James (1977: 532).

##### * Dexopollenia maculata

Taxon classificationAnimaliaDipteraCalliphoridae

Villeneuve, 1933

###### Materials.

1♂, Nantou County, Hohuan-shan, Tayulin, 2700 m, 23.vii.1985, S. Shinonaga (NSMT).

### Subfamily CHRYSOMYINAE

#### Tribe Chrysomyini

##### *Achoetandrus* Bezzi, 1927

###### Achoetandrus rufifacies

Taxon classificationAnimaliaDipteraCalliphoridae

(Macquart, 1843)

####### Materials.

Orchid Is.: 28♂ 59♀, Taitung County, Lanyu Township, Hongtou, Weather Station, 231 m, tropical rainforest, 01.vi.2013, S. T. Yang (NTU); Orchid Is.: 1♂ 9♀, Taitung County, Lanyu Township, Hongtou, 15 m, seashore, 01.vi.2013, S. T. Yang (NTU); Orchid Is.: 8♂ 9♀, Taitung County, Lanyu Township, Longmen Harbor, 5 m, seashore, 01.vi.2013, S. T. Yang (NTU); Orchid Is.: 4♂ 1♀, Taitung County, Lanyu Township, Dongqing, Qingrendong, 18 m, seashore, 01.vi.2013, S. T. Yang (NTU); Orchid Is.: 1♀, Taitung County, Lanyu Township, Zhong'ai Bridge, 53 m, tropical rainforest, 01.vi.2013, S. T. Yang (NTU); 10♂ 35♀, Chiayi County, Alishan Township, Shanmei, Tanayiku, 450 m, secondary forest, 24.ii.2013, S. T. Yang (NTU); 1♂ 2♀, Kaohsiung City, Jiaxian Dist., Jiaxian, 278 m, secondary forest, 12.v.2013, S. T. Yang (NTU); 4♂ 18♀, Taitung County, Haiduan Township, Xiama, 790 m, secondary forest, 30.v.2013, S. T. Yang (NTU); 3♂ 3♀, Taitung County, Haiduan Township, Xinwulu, 390 m, secondary forest, 30.v.2013, S. T. Yang (NTU); 2♂ 12♀, Taitung County, Luye Township, Ruiyuan, 198 m, farmland, 30.v.2013, S. T. Yang (NTU); 1♂, Taitung County, Beinan Township, Jhihben, 209 m, secondary forest, 15.ii.2013, S. T. Yang (NTU); 4♀, Taitung County, Dawu Township, Dawu, 85 m, secondary forest, 18.iv.2013, S. T. Yang (NTU); 7♂ 25♀, Hualien County, Xincheng Township, Qixingtan, Sihba Height, 41 m, grassland, 09.v.2013, S. T. Yang (NTU); 2♀, Hualien County, Xincheng Township, Qixingtan, 14 m, seashore, 09.v.2013, S. T. Yang (NTU); 1♀, Hualien County, Xiulin Township, Fushi, Changuang Temple, 120 m, monsoon rainforest, 09.v.2013, S. T. Yang (NTU); 2♀, Hualien County, Shoufeng Township, Pinghe, 41 m, plain, 16.ii.2013, S. T. Yang (NTU); 1♀, Hualien County, Yuli Township, 133 m, plain, 16.ii.2013, S. T. Yang (NTU); 4♂ 13♀, Pingtung County, Sangdimen Township, Sandi Village, 381 m, secondary forest, 2.iv.2013, S. T. Yang (NTU); 2♂, Pingtung County, Manzhou Township, Qikong Waterfall, 40-125 m, secondary forest, 24.i.2013, S. T. Yang (NTU); Liuqiu Is.: 2♂ 21♀, Pingtung County, Liuqiu Township, Geban Bay, 10 m, seashore, 04.v.2013, S. T. Yang (NTU); Liuqiu Is.: 1♂ 3♀, Pingtung County, Liuqiu Township, Shanfu, 25 m, seashore, 04.v.2013, S. T. Yang (NTU); 1♀, Pingtung County, Shizi Township, Shuangliu, 352 m, secondary forest, 19.v.2013, S. T. Yang (NTU); 2♀, Taipei City, Da'an Dist., Fuzhoushan Park, 71 m, secondary forest, 17.iii.2013, S. T. Yang (NTU); 7♀, Taipei City, Da'an Dist., Fuzhoushan Park, 100 m, secondary forest, 17.iii.2013, S. T. Yang (NTU); 8♂ 20♀, Taipei City, Da'an Dist., NTU Agricultural Entomology Building, 12 m, orchard; eggs collected, 05.ii.2013 eclosion, 19.viii.2009, S. T. Yang (NTU); 1♀, Taipei City, Wenshan Dist., Saint's Alp, 144 m, secondary forest, 21.xii.2012, S. T. Yang (NTU); 3♀, Taipei City, Neihu Dist., Daluntou Mt., 352 m, secondary forest, 19.v.2013, S. T. Yang (NTU); 1♀, Yilan County, Toucheng Township, Beiguan, 34 m, seashore, 04.iv.2013, S. T. Yang (NTU); 1♀, Kaohsiung, Shanping, 640 m, 21–30.iv.1988, C. Young, R. Davidson & J. Rawlins (CMNH); 1♂ 3♀, Puli, Nanshanhsi, 23-24.v.1992, H. M. Lin (NSMT); 2♂ 2♀, Taitung, Chihpen Hot Spring, 3-4.vi.1992, H. M. Lin (NSMT); 1♂ 4♀, Fushan B. G., 600 m, 3–6.v.2006, H. Kurahashi (NSMT); 1♂ 1♀, Chihpen, 15.XI.1985, K. Kanmiya (NSMT); 1♂ 5♀, Antung, 12.XI.1985, K. Kanmiya (NSMT); 2♀, Tainan, Kuantzling, 26.v.1992, R. Kano (NSMT); 1♀, Nanshanchi, 1.v.2006, H. Kurahashi (NSMT); 5♀, Liuknei, 12.VII.1985, S. Shinonaga (NSMT); 1♂ 3♀, Kenting Park, 18-19.XI.1985, K. Kanmiya, R. Kano & H. M. Lin (NSMT); 2♂ 1♀, Chihpen, 15.XI.1985, K. Kanmiya, R. Kano & H. M. Lin (NSMT); 2♂ 3♀, Santimen, 13.VII.1985, S. Shinonaga (NSMT); 1♀, Kuan-zu-ling, 250m, 6.IV.1965, R. Kano (NSMT); 1♀, Chiai, 7.IV.1965, C. M. Yoshimoto & B. D. Parkins (NSMT); 2♂ 8♀, Antung, 12.XI.1985, K. Kanmiya, R. Kano & H. M. Lin (NSMT).

###### Achoetandrus villeneuvi

Taxon classificationAnimaliaDipteraCalliphoridae

(Patton, 1922)

####### Materials.

1♂ 5♀, Taitung County, Beinan Township, Jhihben, 209 m, secondary forest, 15.ii.2013, S. T. Yang (NTU); 2♀, Hualien County, Xiulin Township, Fushi, Changuang Temple, 120 m, monsoon rainforest,09.v.2013, S. T. Yang (NTU); 1♀, Hualien County, Wanrong Township, Wanrong, 1000 m, secondary forest, 08.iv.2013, W. H. Lin (NTU); 2♀, Chiayi County, Alishan Township, Shanmei, Tanayiku, 450 m, secondary forest, 24.ii.2013, S. T. Yang (NTU); 4♀, Taitung County, Haiduan Township, Xiama, 790 m, secondary forest, 30.v.2013, S. T. Yang (NTU); 2♀, Taipei City, Neihu Dist., Daluntou Mt., 352 m, secondary forest, 19.v.2013, S. T. Yang (NTU); 2♂, Luchan, 1,000 m, 24-25.vii.1985, S. Shinonaga (NSMT); 6♂ 2♀, Fushan B. G., 600 m, 3–6.v.2006, H. Kurahashi (NSMT); 2♂ 4♀, Kaohsiung, Shanping, 640 m, 21-30.iv.1988, C. Young, R. Davidson & J. Rawlins (CMNH); 1♂, Taoyuen, Paling, 9-11.vi.1992, R. Kano (NSMT); 1♂ 1♀, Nanshanchi, 1.v.2006, H. Kurahashi (NSMT); 1♂, Ten-chih, 23-04-03 N, 120-45-13 E, 1,550 m, 23.viii.1996, C. W. Young (CMNH).

##### *Ceylonomyia* Fan, 1965

###### * Ceylonomyia nigripes

Taxon classificationAnimaliaDipteraCalliphoridae

(Aubertin, 1932)

####### Materials.

Orchid Is.: 10♂ 16♀, Taitung County, Lanyu Township, Hongtou, Weather Station, 231 m, tropical rainforest, 01.vi.2013, S. T. Yang (NTU); Orchid Is.: 1♂ 1♀, Taitung County, Lanyu Township, Longmen Harbor, 5 m, seashore, 01.vi.2013, S. T. Yang (NTU); Orchid Is.: 1♀, Taitung County, Lanyu Township, Hongtou, 15 m, seashore, 01.vi.2013, S. T. Yang (NTU); 1♀, Hualien County, Xiulin Township, Fushi, Changuang Temple, 120 m, monsoon rainforest, 09.v.2013, S. T. Yang (NTU); 1♀, Kaohsiung City, Jiaxian Dist., Jiaxian, 278 m, secondary forest, 12.v.2013, S. T. Yang (NTU); 1♂, Fenchifu, 20.XI.1985, K. Kanmiya (NSMT); 12♂ 21♀, Kenting Park, 18–19.XI.1985, K. Kanmiya (NSMT); 2♂, Antung, 12.XI.1985, K. Kanmiya (NSMT); LANHSU IS.: 1♂ 1♀, 17–19.VII.1985, S. Shinonaga (NSMT); 1♂ 5♀, Santimen, 13.VII.1985, S. Shinonaga (NSMT); 10♂ 3♀, Liuknei, 12.VII.1985, S. Shinonaga (NSMT); 1♀, Kenting Park, 18–19.xi.1985, K. Kanmiya (NSMT); 3♀, Fushan B. G., 600 m, 3–6.v.2006, H. Kurahashi (NSMT); 1♂, Kaohsiung, Shanping, 640 m, 21–30.iv.1988, C. Young, R. Davidson & J. Rawlins (CMNH); 1♀, Juisui, 10.XI.1985, K. Kanmiya, R. Kano & H. M. Lin (NSMT); 1♀, Juisui, 11.XI.1985, K. Kanmiya (NSMT); 3♂, Kenting Park, 18-19.XI.1985, K. Kanmiya, R. Kano & H. M. Lin (NSMT); 1♀, Kenting Park, 18-19.xi.1985, K. Kanmiya (NSMT).

##### *Chrysomya* Robineau-Desvoidy, 1830

###### Chrysomya bezziana

Taxon classificationAnimaliaDipteraCalliphoridae

Villeneuve, 1914

####### Note.

See to Fan (1992: 544).

###### Chrysomya megacephala

Taxon classificationAnimaliaDipteraCalliphoridae

(Fabricius, 1794)

####### Materials.

65♂ 87♀, Hualien County, Xincheng Township, Qixingtan, Sihba Height, 41 m, grassland, 09.v.2013, S. T. Yang (NTU); 4♂ 6♀, Hualien County, Xincheng Township, Qixingtan, 14 m, seashore, 09.v.2013, S. T. Yang (NTU); 1♀, Hualien County, Xiulin Township, Taroko, 81 m, canyon, 09.v.2013, S. T. Yang (NTU); 1♀, Hualien County, Guangfu Township, 143 m, plain,16.ii.2013, S. T. Yang (NTU); 2♂ 20♀, Hualien County, Shoufeng Township, Pinghe, 41 m, plain, 16.ii.2013, S. T. Yang (NTU); 2♂ 5♀, Taipei City, Neihu Dist., Daluntou Mt., 352 m, secondary forest, 19.v.2013, S. T. Yang (NTU); 1♂ 19♀, Taipei City, Da'an Dist., Fuzhoushan Park, 71 m, secondary forest, 17.iii.2013, S. T. Yang (NTU); 1♂ 1♀, Taipei City, Da'an Dist., NTU Agricultural Entomology Building, 12 m, orchard, 22.i.2013, S. T. Yang (NTU); 1♀, New Taipei City, Shimen Dist., Linshan Cape, 4 m, seashore, 21.xii.2012, S. T. Yang (NTU); 12♀, New Taipei City, Shimen Dist., Linshan Cape, 4 m, seashore, 29.xii.2012, S. T. Yang (NTU); 1♂, New Taipei City, Wulai Dist., Hsiaoi Village, Tonghou, 233 m, riverside, 27.xii.2012, S. T. Yang (NTU); 1♀, New Taipei City, Xindian Dist., Zhitan Dam, 57 m, riverside, 27.xii.2012, S. T. Yang (NTU); 1♂ 20♀, Taitung County, Luye Township, Ruiyuan, 198 m, farmland, 30.v.2013, S. T. Yang (NTU); 1♀, Taitung County, Beinan Township, Jhihben, 209 m, secondary forest, 15.ii.2013, S. T. Yang (NTU); 3♂ 31♀, Taitung County, Dawu Township, Dawu, 85 m, secondary forest, 18.iv.2013, S. T. Yang (NTU); 7♂ 3♀, Taitung County, Haiduan Township, Xiama, 790 m, secondary forest, 30.v.2013, S. T. Yang (NTU); 1♂, Taitung County, Haiduan Township, Xinwulu, 390 m, secondary forest, 30.v.2013, S. T. Yang (NTU); Orchid Is.: 13♂ 59♀, Taitung County, Lanyu Township, Hongtou, Weather Station, 231 m, tropical rainforest, 01.vi.2013, S. T. Yang (NTU); Orchid Is.: 3♂ 18♀, Taitung County, Lanyu Township, Hongtou, 15 m, seashore, 01.vi.2013, S. T. Yang (NTU); Orchid Is.: 2♀, Taitung County, Lanyu Township, Yeyou, 8 m, seashore, 31.v.2013, S. T. Yang (NTU); Orchid Is.: 2♀, Taitung County, Lanyu Township, Longmen Harbor, 5 m, seashore, 01.vi.2013, S. T. Yang (NTU); Mazu, Beigan Is.: 4♂ 2♀, Lienchiang County, Beigan Township, 100 m, seashore, 12.v.2013, Y. C. Yu (NTU); Liuqiu Is.: 6♂ 33♀, Pingtung County, Liuqiu Township, Shanfu, 25 m, seashore, 04.v.2013, S. T. Yang (NTU); Liuqiu Is.: 6♂ 5♀, Pingtung County, Liuqiu Township, Shanfu, 25 m, seashore; eggs collected, 17.v.2013 eclosion, 04.v.2013, S. T. Yang (NTU); Liuqiu Is.: 6♂ 7♀, Pingtung County, Liuqiu Township, Dafu Fishing Port, 8 m, seashore; eggs collected, 17.v.2013 eclosion, 04.v.2013, S. T. Yang (NTU); Liuqiu Is.: 1♂, Pingtung County, Liuqiu Township, Dafu Fishing Port, 8 m, seashore, 04.v.2013, S. T. Yang (NTU); Liuqiu Is.: 1♂ 4♀, Pingtung County, Liuqiu Township, White Lighthouse, 82 m, secondary forest, 04.v.2013, S. T. Yang (NTU); Liuqiu Is.: 2♂ 115♀, Pingtung County, Liuqiu Township, Geban Bay, 10 m, seashore, 04.v.2013, S. T. Yang (NTU); 5♂ 14♀, Pingtung County, Manzhou Township, Qikong Waterfall, 40-125 m, secondary forest, 24.i.2013, S. T. Yang (NTU); 1♂ 16♀, Pingtung County, Sangdimen Township, Sandi Village, 381 m, secondary forest, 02.iv.2013, S. T. Yang (NTU); 3♂ 3♀, Kaohsiung City, Jiaxian Dist., Jiaxian, 278 m, secondary forest, 12.v.2013, S. T. Yang (NTU); 1♂ 2♀, Tainan City, Baihe Dist., Guanziling, Lingding Park, 322 m, secondary forest, 21.iii.2013, S. T. Yang (NTU); 1♂ 2♀, Chiayi County, Alishan Township, Shanmei, Tanayiku, 450 m, secondary forest, 24.ii.2013, S. T. Yang (NTU); 1♂ 3♀, Yilan County, Toucheng Township, Beiguan, 34 m, seashore, 04.iv.2013, S. T. Yang (NTU); 5♀, Fenchifu, 20.XI.1985, K. Kanmiya (NSMT); 2♂, Santimen, 13.VII.1985, S. Shinonaga (NSMT); 1♂, Hori, 2.VII.1965, T. Shirozu (NSMT); 1♂ 1♀, Honbukei, 2.V.1965, T. Shirozu (NSMT); 1♀, Chu-ch'i, 120m, 13.IV.1965, R. Kano (NSMT); 4♂ 5♀, Chichiwanchi, Huanshan, 5-6.XI.1985, K. Kanmiya (NSMT); 1♀, Chihpen, 15.XI.1985, K. Kanmiya (NSMT); 1♀, San Palin, 1,100m, 25.X.1985, R. Kano (NSMT); 2♂ 8♀, Antung, 12.XI.1985, K. Kanmiya (NSMT); 2♂, Yangmin-shan, 28.X.1985, K. Kanmiya (NSMT); 2♀, Juisui, 10.XI.1985, K. Kanmiya (NSMT); LAN HSU IS.: 1♂, Yehyin, 7.VII.1971, K. Mizusawa (NSMT); 1♀, Wulai, 4.VII.1985, S. Shinonaga (NSMT); LANHSU IS.: 4♂ 6♀, 17–19.VII.1985, S. Shinonaga (NSMT); 2♂ 2♀, Liuknei, 12.VII.1985, S. Shinonaga (NSMT); 2♂ 2♀, Fenchifu, 1,400m, 8.VII.1985, S. Shinonaga (NSMT); 3♂, Kenting Park, 15.VII.1985, S. Shinonaga (NSMT); 1♂, Taipei, 30.III.1965, R. Kano (NSMT); 2♂, Hohuan-sahn, Tsuifeng, 2,400m, 23–24.VII.1985, S. Shinonaga (NSMT); 1♂ 3♀, Antung, 12.XI.1985, K. Kanmiya (NSMT); 2♂, Kwantyling, 11.VII.1985, S. Shinonaga (NSMT); 1♀, Taihoku, 18.vii.1927, F. C. Hadden (BPBM); 2♀, Taoyuen, Paling, 9-11.vi.1992, R. Kano (NSMT); 3♀, Kenting Park, 1–2.vi.1992, H. M. Lin (NSMT); 1♂ 2♀, Chung-Hsien University, Huei-sun Forest, 21.v.1992, R. Kano (NSMT); 6♂ 18♀, Ten-chih, 23-04-03 N, 120-45-13 E, 1,550 m, 23.viii.1996, C. W. Young (CMNH); 1♂, Kuantzuling, 26.v.1992, R. Kano (NSMT); 5♂ 12♀, Kaohsiung, Shanping, 640 m, 21–30.iv.1988, C. Young, R. Davidson & J. Rawlins (CMNH); 1♀, Taihoku, 18.vii.1927, F. C. Hadden (BPBM); 1♂ 1♀, Fushan B. G., 600 m, 3–6.v.2006, H. Kurahashi (NSMT); 1♂ 1♀, Puli, Nanshahsi, 23–24.v.1992, H. M. Lin (NSMT).

###### Chrysomya pinguis

Taxon classificationAnimaliaDipteraCalliphoridae

(Walker, 1858)

####### Materials.

1♂ 1♀, Nantou County, Ren'ai Township, Aowanda, 18.v.2013, K. Harusawa (PCKHa); 1♀, Nantou County, Ren'ai Township, Gaofeng, Guantou Mt. (Northeast), 20.v.2013, K. Harusawa (PCKHa); 41♂ 119♀, Taitung County, Beinan Township, Jhihben, 209 m, secondary forest, 15.ii.2013, S. T. Yang (NTU); 2♂ 18♀, Taitung County, Haiduan Township, Xiama, 790 m, secondary forest, 30.v.2013, S. T. Yang (NTU); 1♀, New Taipei City, Gongliao Dist., Santiago Cape, 97 m, bushes, 28.ii.2013, S. T. Yang (NTU); 1♂ 7♀, Taipei City, Da'an Dist., Fuzhoushan Park, 71 m, secondary forest, 17.iii.2013, S. T. Yang (NTU); 1♀, Yilan County, Toucheng Township, Beiguan, 34 m, seashore, 04.iv.2013, S. T. Yang (NTU); 1♀, Hualien County, Xiulin Township, Taroko, 81 m, canyon, 09.v.2013, S. T. Yang (NTU); 3♀, Hualien County, Xiulin Township, Fushi, Changuang Temple, 120 m, monsoon rainforest, 09.v.2013, S. T. Yang (NTU); Mazu, Beigan Is.: 1♂, Lienchiang County, Beigan Township, 100 m, seashore, 12.v.2013, Y. C. Yu (NTU); 3♀, New Taipei City, Xindian Dist., Qingtan, Sishifen, 419 m, secondary forest, 07.ii.2013, S. T. Yang (NTU); 44♂ 44♀, Taipei City, Neihu Dist., Daluntou Mt., 352 m, secondary forest, 19.v.2013, S. T. Yang (NTU); 1♂, Pingtung County, Manzhou Township, Qikong Waterfall, 40–125 m, secondary forest, 24.i.2013, S. T. Yang (NTU); 4♀, Pingtung County, Shizi Township, Shuangliu, 189-300 m, secondary forest, 11.v.2013, S. T. Yang (NTU); 27♂ 24♀, Nantou County, Ren'ai Township, Meifeng Farm, 2100 m, secondary forest; eggs collected, 23.i.2013 eclosion, 28.viii.2011, Y. J. Liu (NTU); 9♂ 19♀, Nantou County, Lugu Township, Xitou, 1156 m, secondary forest, 08.iii.2013, S. T. Yang (NTU); 1♂ 5♀, Nantou County, Ren'ai Township, Songgang, 2049 m, secondary forest, 16.iv.2013, S. T. Yang (NTU); 2♀, New Taipei City, Wugu Dist., Guanyin Mt., Yinghan Peak, 359-611 m, secondary forest, 30.iv.2013, S. T. Yang (NTU); 4♂ 9♀, Taichung City, Heping Dist., Huanshan Tribe, Ssuchiehlan Stream, 1832 m, riverbed; larvae collected, 09.ii.2013 eclosion, 26.i.2013, S. T. Yang (NTU); 39♂ 94♀, New Taipei City, Wulai Dist., Xinxian Village, 219 m, riverside, 16.iii.2013, S. T. Yang (NTU); 3♂ 16♀, Taipei City, Wenshan Dist., Saint's Alp, 144 m, secondary forest, 21.xii.2012, S. T. Yang (NTU); 5♂ 1♀, Yilan County, Toucheng Township, Yingzi Mt., 936 m, bush, 27.v.2013, S. T. Yang (NTU); 4♀, Chiayi County, Alishan Township, Shanmei, Tanayiku, 450 m, secondary forest, 24.ii.2013, S. T. Yang (NTU); 1♀, New Taipei City, Shimen Dist., Linshan Cape, 20 m, seashore, 21.xii.2012, S. T. Yang (NTU); 7♂ 12♀, New Taipei City, Wulai Dist., Hsiaoi Village, Tonghou, 233 m, riverside, 27.xii.2012, S. T. Yang (NTU); 4♀, New Taipei City, Xindian Dist., Zhitan Dam, 57 m, riverside, 27.xii.2012, S. T. Yang (NTU); 1♀, New Taipei City, Wulai Dist., Zhongzhi, 109 m, riverside, 27.xii.2012, S. T. Yang (NTU); 2♂ 6♀, New Taipei City, Ruifang Dist., Jinguashih, Chahu Mt., 343 m, bush, 01.ii.2013, S. T. Yang (NTU); 1♂ 11♀, New Taipei City, Ruifang Dist., Jinguashih, Chahu Mt., 416 m, bush, 01.ii.2013, S. T. Yang (NTU); 1♀, New Taipei City, Ruifang Dist., Jinguashih, 360 m, house, 05.ii.2013, T. H. Wu (NTU); 1♂, San Palin, 1,100m, 25.X.1985, R. Kano (NSMT); 3♂ 2♀, Lalashan, Taoyuen, 12.vi.1992, H. M. Lin (NSMT); 2♀, Chihpen, 15.XI.1985, K. Kanmiya, R. Kano & H. M. Lin (NSMT); 6♂ 5♀, Huanshan, Chichiawanchi, 5-6.XI.1985, K. Kanmiya, R. Kano & H. M. Lin (NSMT); 1♂, Lafu, 350m, 27.X.1985, R. Kano (NSMT); 2♂ 7♀, Fenchifu, 20.XI.1985, K. Kanmiya, R. Kano & H. M. Lin (NSMT); 2♂ 6♀, Lala shan, 1,400-1,600m, 25–26.X.1985, R. Kano & H. M. Lin (NSMT); 2♂, Antung, 12.XI.1985, K. Kanmiya, R. Kano & H. M. Lin (NSMT); 3♂ 2♀, Hohuan-shan, Yuankang, 2,700m, 23.VII.1985, S. Shinonaga (NSMT); 2♀, Chiapaotai, 4.XI.1985, K. Kanmiya, R. Kano & H. M. Lin (NSMT); 1♂ 1♀, Mt. Yang-ming Shan, 450m, 28.III.1965, R. Kano (NSMT); 1♂ 1♀, Lushan, 1,000m, 24–25.VII.1985, S. Shinonaga (NSMT); 1♀, Taichung, Pilushi, 2,200 m, 22-23.v.1988, R. Davidson, C. Young & J. Rawlins (CMNH); 4♀, Taitung, Chihpen Hot Spring, 3–4.vi.1992, H. M. Lin (NSMT); 4♂ 3♀, Taoyuen, Paling, 9-11.vi.1992, R. Kano (NSMT); 8♂ 4♀, Puli, Nanshanhsi, 23–24.v.1992, N. M. Lin (NSMT); 1♂ 5♀, Chung-Hsien University, Huei-sun Forest, 21.v.1992, R. Kano (NSMT); 1♂ 1♀, Alishan, 2,400m, 9-10.VII.1985, S. Shinonaga (NSMT); 4♂ 4♀, Mt. Alishan, 28–29.v.1992, R. Kano (NSMT); 22♂ 33♀, Kaohsiung, Shanping, 640 m, 21–30.iv.1988, C. Young, R. Davidson & J. Rawlins (CMNH); 2♂, Wulai, Taipei, 15.vi.1992, R. Kano (NSMT); 1♂, Anmashan, 2,230 m, 30.iv.–4.v.1990, A. Smetana (CMNH); 3♂ 7♀, Nanshanchi, 1.v.2006, H. Kurahashi (NSMT); 3♂ 7♀, nr Huanshan, 2.v.2006, H. Kurahashi (NSMT); 2♂ 3♀, Ten-chih, 23-04-03 N, 120-45-13 E, 1,550 m, 23.viii.1996, C. W. Young (CMNH); 17♂ 21♀, Fushan B. G., 600 m, 3–6.v.2006, H. Kurahashi (NSMT); 18♂ 4♀, Hohuan-sahn, Tsuifeng, 2,400m, 23–24.VII.1985, S. Shinonaga (NSMT).

#### Tribe Phormiini

##### *Protocalliphora* Hough, 1899

###### Protocalliphora sp.

Taxon classificationAnimaliaDipteraCalliphoridae

####### Materials.

1♀, Mt. Alishan, 2,400 m, 9-10.vii.1985, S. Shinonaga (NSMT). [See to Kurahashi (2000: 27)]

### Subfamily RHINIINAE

#### Tribe Rhiniini

##### *Idiella* Brauer & Bergenstamm, 1889

###### * Idiella divisa

Taxon classificationAnimaliaDipteraCalliphoridae

(Walker, 1861)

####### Materials.

4♂ 5♀, Chihpen, 15.xi.1985, K. Kanmiya (NSMT); 1♂, Shui-tao, 220 m, 15.iv.1915, S. Ueno (NSMT); 1♀, Fenchifu, 20.xi.1965, K. Kanmiya (NSMT).

###### Idiella euidielloides

Taxon classificationAnimaliaDipteraCalliphoridae

Senior-White, 1923

####### Materials.

2♂, Nantou County, Ren'ai Township, Aowanda, 18.v.2013, K. Harusawa (PCKHa); 2♂ 1♀, Nantou County, Ren'ai Township, Gaofeng, Guantou Mt. (Northeast), 20.v.2013, K. Harusawa (PCKHa); 1♀, Chihpen, 15.xi.1985, K. Kanmiya (NSMT); 1♂, Chung-Hsien University, Huei-sun Forest, 21.v.1992, R. Kano (NSMT); 2♂ 4♀, Taoyuen, Paling, 9-11.vi.1992, R. Kano (NSMT); 3♀, Nanshanhsi, nr Puli, 23–24.v.1992, R. Kano & H. M. Lin (NSMT); 1♀, Meifeng, 2,130 m, 10–17.vii.1993, A. Smetana (CMNH); 1♀, Kaohsiung, Ten-chih, 23.04'03"N, 120.45'13"E, 1,550 m, 23.viii.1996, C. W. Young (CMNH); 1♀, Chihpen, 15.xi.1985, K. Kanmiya (NSMT); 8♂ 3♀, Fushan B. G., 600 m, 3-6.v.2006, H. Kurahashi (NSMT); 1♂ 1♀, Chihpen, 15.xi.1985, K. Kanmiya (NSMT); 1♀, Hsitou, 1,000 m, 1.v.2006, H. Kurahashi (NSMT); 3♀, Nanshanchi, 1.v.2006, H. Kurahashi (NSMT).

###### Idiella mandarina

Taxon classificationAnimaliaDipteraCalliphoridae

(Wiedemann, 1830)

####### Materials.

1♂, Kenting-kung-yuan, 3.iv.1965, R. Kano (NSMT).

##### *Rhinia* Robineau-Desvoidy, 1830

###### Rhinia apicalis

Taxon classificationAnimaliaDipteraCalliphoridae

(Wiedemann, 1830)

####### Materials.

1♀, Nantou County, Ren'ai Township, Lushan, 1000 m, 10.x.2010, S. T. Yang (NTU); 1♂, Hohuan-shan, Kunyan, 2,700 m, 23.vii.1985, S. Shinonaga (NSMT); 2♀, Hohuan-shan, Tsuifeng, 2,400 m, 23-24.vii.1985, S. Shinonaga (NSMT); 1♀, Lushan, 1,000 m, 24-25.vii.1985, S. Shinonaga (NSMT); 1♂ 4♀, Huanshan, Chichiawanchi, 6.xii.1985, K. Kanmiya (NSMT); 1♂, Tsuifeng-Shunkan, 24.vii.1985, H. Shima (NSMT); 1♀, Nanshanhsi nr Puli, 23-24.v.1992, R. Kano (NSMT); 1♂, Hohuan-shan, Tsuifeng-Shunkan, 24.vii.1985, H. Shima (NSMT); 1♂, Tsuifeng-Shunkan, 24.vii.1985, H. Shima (NSMT).

###### Rhinia sauteri

Taxon classificationAnimaliaDipteraCalliphoridae

Peris, 1951

####### Note.

See to James (1977: 553).

##### *Stomorhina* Rondani, 1861

###### Stomorhina discolor

Taxon classificationAnimaliaDipteraCalliphoridae

(Fabricius, 1794)

####### Materials.

1♀, Pingtung County, Manzhou Township, Qikong Waterfall, 40-125 m, secondary forest, 24.i.2013, S. T. Yang (NTU); 1♂, Kentin, 1.II.1972, K. Matsuki (NSMT); 1♂, Kentin Park (Kontei Park), 13.viii.1980, K. Hara (NSMT); 2♀, Antung, 12.x.1985, K. Kanmiya (NSMT); 1♂, Juisui, 10.xi.1985, K. Kanmiya (NSMT); 1♀, Oluanpi (Galanpi), 13.viii.1980, K. Hara (NSMT); 1♂, nr Alishan, Shanmei, 1,300 m, 29.iv.2006, H. Kurahashi (NSMT); 1♂, Ch'-hsin-liao, 15.iv.1965, S. Ueno (NSMT); 1♂, Lushan, 1,000 m, 24-25.vii.1985, S. Shinonaga (NSMT).

###### Stomorhina lunata

Taxon classificationAnimaliaDipteraCalliphoridae

(Fabricius, 1805)

####### Note.

See to Hennig (1941: 181).

###### * Stomorhina obsoleta

Taxon classificationAnimaliaDipteraCalliphoridae

(Wiedemann, 1830)

####### Materials.

1♀, Lushan, 1,000 m, 24-25.vii.1985, S. Shinonaga (NSMT); 1♀, Fen-ch'i-hu, 12.iv.1965, R. Kano (NSMT); 1♂, Hotso (Roshan), 30.ix.1965, K. Kaneko (NSMT).

###### Stomorhina veterana

Taxon classificationAnimaliaDipteraCalliphoridae

Villeneuve, 1927

####### Materials.

1♂, Yingfeng (Gokansan), 19.viii.1980, K. Hara (NSMT); 1♀, Alishan-Yushan, 2,600-2,700 m, 31.x.1985, M. Iwasa (NSMT); 1♀, Hohuan-shan, Tsuifeng, 2,400 m, 23-24.vii.1985, S. Shinonaga (NSMT); 1♀, Hohuan-shan, Kunyan, 2,700 m, 23.vii.1985, S. Shinonaga (NSMT); 7♀, Meifeng, 2,130 m, 10-17.vii.1993, A. Smetana (CMNH); 1♂, Fenchihu, 4.iv.1965, R. Kano (NSMT); 1♀, Ten-chin, 23.04'03"N 120.45'13"E, 1,550 m, 23.viii.1996, C. W. Young (CMNH); 1♀, Tattaka, 10.iv.1965, T. Saigusa (NSMT); 1♂, Mt. Yui-shan, 2,700-3,500 m, 6-7.vii.1985, S. Shinonaga (NSMT); 1♀, Mt. Yui-shan, 2,700-3,500m, 6-7.VII.1985, S. Shinonaga (NSMT); 1♀, Hohuan-shan, Yuankang, 2,700m, 23.VII.1985, S. Shinonaga (NSMT); 12♀, Hohuan-shan, Tsuifeng, 2,400m, 23-24.VII.1985, S. Shinonaga (NSMT); 1♂, Fenchihu, 12.IV.1965, R. Kano (NSMT); 1♂ 1♀, Tatachia-anpu, 31.x.1985, K. Kanmiya (NSMT); 1♂, Tatachiaanpu-Paiyunshanchuan, 6.vii.1985, H. Shima (NSMT); 1♀, Alishan-Yushan, 2,600-2,700 m, 31.x.1985, M. Iwasa (NSMT); 1♀, Alishan-Yushan, 2,600-2,700 m, 31.x.1985, M. Iwasa (NSMT).

###### Stomorhina xanthogaster

Taxon classificationAnimaliaDipteraCalliphoridae

(Wiedemann, 1820)

####### Materials.

9♂ 4♀, New Taipei City, Shimen Dist., Linshan Cape, 20 m, seashore, 29.xii.2012, S. T. Yang (NTU).

#### Tribe Cosminini

##### *Borbororhinia* Townsend, 1917

###### Borbororhinia bivittata

Taxon classificationAnimaliaDipteraCalliphoridae

(Walker, 1857)

####### Note.

See to James (1977: 544).

##### *Isomyia* Walker, 1860

###### * Isomyia delectans

Taxon classificationAnimaliaDipteraCalliphoridae

(Walker, 1860)

####### Materials.

1♀, Yilan County, Toucheng Township, Yingzi Mt., 936 m, bush, 27.v.2013, S. T. Yang (NTU).

###### Isomyia electa

Taxon classificationAnimaliaDipteraCalliphoridae

(Villeneuve, 1927)

####### Materials.

1♂, New Taipei City, Wulai Dist., Wulai, 500 m, secondary forest, 20.vi.2011, Y. C. Yu (NTU); 2♂ 8♀, Fushan B. G., 600 m, 3-6.v.2006, H. Kurahashi (NSMT).

###### * Isomyia oestracea

Taxon classificationAnimaliaDipteraCalliphoridae

(Séguy, 1934)

####### Materials.

1♀, Taitung County, Haiduan Township, Xiama, 790 m, secondary forest, 30.v.2013, S. T. Yang (NTU); 4♂, Fushan B. G., 600 m, 3-6.v.2006, H. Kurahashi (NSMT); 1♀, Fushan Botanical Garden, 750 m, 28.vi.2010, K. Harusawa (PCKHa).

###### * Isomyia pseudolucilia

Taxon classificationAnimaliaDipteraCalliphoridae

(Malloch, 1928)

####### Materials.

2♂ 1♀, Nantou County, Ren'ai Township, Aowanda, 18.v.2013, K. Harusawa (PCKHa); 1♂ 1♀, New Taipei City, Wugu Dist., Guanyin Mt., Yinghan Peak, 359–611 m, secondary forest, 30.iv.2013, S. T. Yang (NTU); 3♀, Taipei City, Neihu Dist., Daluntou Mt., 352 m, secondary forest, 19.v.2013, S. T. Yang (NTU); 2♀, Liuknei, 12.vii.1985, S. Shinonaga (NSMT); 17♂ 37♀, Fushan B. G., 600 m, 3-6.v.2006, H. Kurahashi (NSMT).

###### Isomyia tibialis

Taxon classificationAnimaliaDipteraCalliphoridae

(Villeneuve, 1927)

####### Materials.

1♂, Kukan, 3.xi.1985, K. Kanmiya (NSMT); 1♀, Tsuifen-Shunkan, 24.vii.1985, H. Shima (NSMT); 1♀, Paling, Taoyuen, 9–11.vi.1992, R. Kano (NSMT).

###### Isomyia viridaurea

Taxon classificationAnimaliaDipteraCalliphoridae

(Wiedemann, 1819)

####### Note.

See to James (1977: 551).

##### *Rhyncomya* Robineau-Desvoidy, 1830

###### Rhyncomya notata

Taxon classificationAnimaliaDipteraCalliphoridae

(van Der Wulp, 1880)

####### Note.

See to Fan (1992: 565).

###### Rhyncomya setipyga

Taxon classificationAnimaliaDipteraCalliphoridae

Villeneuve, 1927

####### Materials.

2♂ 1♀, New Taipei City, Shimen Dist., Linshan Cape, 20 m, seashore, 29.xii.2012, S. T. Yang (NTU).

##### *Strongyloneura* Bigot, 1886

###### * Strongyloneura diploura

Taxon classificationAnimaliaDipteraCalliphoridae

Fang & Fan, 1984

####### Materials.

Kinmen Is.: 2♂ 2♀, Shiahsintsuoh, 8.viii.1993, C. L. Chung (NSMT).

###### Strongyloneura prasina

Taxon classificationAnimaliaDipteraCalliphoridae

Bigot, 1886

####### Note.

See to James (1977: 555).

###### * Strongyloneura prolata

Taxon classificationAnimaliaDipteraCalliphoridae

(Walker, 1860)

####### Materials.

1♂, Kuantzuling, 26.v.1992, R. Kano (NSMT).

##### *Sumatria* Malloch, 1926

###### * Sumatria chiekoae

Taxon classificationAnimaliaDipteraCalliphoridae

Kurahashi & Tumrasvin, 1992

####### Materials.

1♂, Lenai, 1,000 m, 23.vii.1985, S. Shinonaga (NSMT); 1♀, Lala Shan, 1,400 m, 25.x.1985, R. Kano (NSMT); 1♂, Lenai, 1,000 m, 23.vii.1985, S. Shinonaga (NSMT); 2♀, Taoyuen, Paling, 9-11.vi.1992, R. Kano (NSMT).

###### Sumatria flava

Taxon classificationAnimaliaDipteraCalliphoridae

(Villeneuve, 1927)

####### Materials.

1♀, Hsitou, 1,000 m, 1.v.2006, H. Kurahashi (NSMT); 1♀, Hsitou, 1,000 m, 1.v.2006, H. Kurahashi (NSMT).

###### * Sumatria vittata

Taxon classificationAnimaliaDipteraCalliphoridae

(Peris, 1952)

####### Materials.

1♂, San Palin, 1,500 m, 26.x.1985, M. Iwasa (NSMT); 1♂, Sai Palin, 1,500 m, 25.x.1985, M. Iwasa (NSMT); 1♂, Sai Palin, 1,500 m, 25.x.1985, M. Iwasa (NSMT).

## Supplementary Material

XML Treatment for Paradichosia lui

XML Treatment for Silbomyia hoeneana

XML Treatment for Silbomyia cyanea

XML Treatment for Silbomyia sauteri

XML Treatment for Silbomyia hoeneana

XML Treatment for Catapicephala dasyophthalma

XML Treatment for Catapicephala ruficornis

XML Treatment for Catapicephala splendens

XML Treatment for Aldrichina grahami

XML Treatment for Bellardia menechma

XML Treatment for Bellardia pubescens

XML Treatment for Calliphora nigribarbis

XML Treatment for Calliphora pattoni

XML Treatment for Calliphora vomitoria

XML Treatment for Polleniopsis dalatensis

XML Treatment for Polleniopsis toxopei

XML Treatment for Tainanina pilisquama

XML Treatment for Tainanina sarcophagoides

XML Treatment for Gymnadichosia pusilla

XML Treatment for Paradichosia crinitarsis

XML Treatment for Paradichosia lui

XML Treatment for Pollenomyia sinensis

XML Treatment for Tricycleopsis paradoxa

XML Treatment for Bengalia calilungae

XML Treatment for Bengalia chekiangensis

XML Treatment for Bengalia emarginata

XML Treatment for Bengalia escheri

XML Treatment for Bengalia fuscipennis

XML Treatment for Bengalia taksina

XML Treatment for Bengalia torosa

XML Treatment for Bengalia varicolor

XML Treatment for Hemipyrellia ligurriens

XML Treatment for Lucilia bazini

XML Treatment for Lucilia calviceps

XML Treatment for Lucilia cuprina

XML Treatment for Lucilia hainanensis

XML Treatment for Lucilia papuensis

XML Treatment for Lucilia porphyrina

XML Treatment for Lucilia sinensis

XML Treatment for Lucilia sericata

XML Treatment for Lucilia taiwanica

XML Treatment for Caiusa indica

XML Treatment for Caiusa testacea

XML Treatment for Caiusa sp.

XML Treatment for Dexopollenia flava

XML Treatment for Dexopollenia luteola

XML Treatment for Dexopollenia maculata

XML Treatment for Achoetandrus rufifacies

XML Treatment for Achoetandrus villeneuvi

XML Treatment for Ceylonomyia nigripes

XML Treatment for Chrysomya bezziana

XML Treatment for Chrysomya megacephala

XML Treatment for Chrysomya pinguis

XML Treatment for Protocalliphora sp.

XML Treatment for Idiella divisa

XML Treatment for Idiella euidielloides

XML Treatment for Idiella mandarina

XML Treatment for Rhinia apicalis

XML Treatment for Rhinia sauteri

XML Treatment for Stomorhina discolor

XML Treatment for Stomorhina lunata

XML Treatment for Stomorhina obsoleta

XML Treatment for Stomorhina veterana

XML Treatment for Stomorhina xanthogaster

XML Treatment for Borbororhinia bivittata

XML Treatment for Isomyia delectans

XML Treatment for Isomyia electa

XML Treatment for Isomyia oestracea

XML Treatment for Isomyia pseudolucilia

XML Treatment for Isomyia tibialis

XML Treatment for Isomyia viridaurea

XML Treatment for Rhyncomya notata

XML Treatment for Rhyncomya setipyga

XML Treatment for Strongyloneura diploura

XML Treatment for Strongyloneura prasina

XML Treatment for Strongyloneura prolata

XML Treatment for Sumatria chiekoae

XML Treatment for Sumatria flava

XML Treatment for Sumatria vittata
